# Prognostic Value of Serum S100B Protein for Neurological Outcomes After Cardiac Arrest: A Systematic Review and Meta-Analysis

**DOI:** 10.3390/jcm15010238

**Published:** 2025-12-28

**Authors:** Łukasz Szpinda, Michal Lis, Michal Pruc, Weronika Goraj, Iwona Niewiadomska, Maciej Maslyk, Katarzyna Kotfis, Hanno L. Tan, Enrico Baldi, Lukasz Szarpak

**Affiliations:** 1 Department of Anaesthesiology and Intensive Care, Czerniakowski Hospital, 00-739 Warsaw, Poland; 2 Department of Internal Medicine, Endocrinology, Diabetology, Nephrology and Metabolic Diseases, Czerniakowski Hospital, 00-739 Warsaw, Poland; 3 Faculty of Medicine, Lazarski University, 02-662 Warsaw, Poland; 4 Department of Clinical Research and Development, LUXMED Group, 02-678 Warsaw, Poland; 5 Institute of Medical Sciences, Collegium Medicum, The John Paul II Catholic University of Lublin, 20-708 Lublin, Poland; 6 Institute of Psychology, The John Paul II Catholic University of Lublin, 20-950 Lublin, Poland; 7 Institute of Molecular Biology, The John Paul II Catholic University of Lublin, Konstantynow 1i Str., 20-708 Lublin, Poland; 8 Department of Anesthesiology, Division of Anesthesiology Critical Care Medicine, Vanderbilt University Medical Center, Nashville, TN 37232, USA; 9 Department of Anesthesiology, Intensive Care and Pain Management, Pomeranian Medical University in Szczecin, 70-110 Szczecin, Poland; 10 Department of Experimental Cardiology, Amsterdam University Medical Center AMC, University of Amsterdam, 1105 AZ Amsterdam, The Netherlands; 11 Division of Cardiology, Fondazione IRCCS Policlinico San Matteo, 27100 Pavia, Italy; 12 Cardiac Arrest and Resuscitation Science Research Team (RESTART), Fondazione IRCCS Policlinico San Matteo, 27100 Pavia, Italy; 13 Technology Transfer Center, The John Paul II Catholic University of Lublin, 20-708 Lublin, Poland; 14 Henry JN Taub Department of Emergency Medicine, Baylor College of Medicine, Houston, TX 77030, USA

**Keywords:** cardiac arrest, heart arrest, S100B calcium binding protein B, biomarkers, neuroprognostication, hypoxic–ischemic brain injury, systematic review, meta-analysis, resuscitation, prognosis

## Abstract

**Background/Objectives**: Cardiac arrest (CA) continues to be one of the leading causes of mortality and long-term neurological disability worldwide. Accurate early neuroprognostication after return of spontaneous circulation is essential for guiding post-resuscitation care. The calcium-binding astrocytic protein S100B has been identified as a potential biomarker for hypoxic–ischemic brain injury. This systematic review and meta-analysis assessed the prognostic and diagnostic efficacy of serum S100B in forecasting neurological outcomes after CA. **Methods:** Thorough searches of PubMed, Embase, Scopus, Web of Science, CENTRAL, and CINAHL from their inception to November 2025 uncovered 40 observational studies. **Results:** Pooled analyses employing random-effects models revealed markedly reduced S100B concentrations in patients with favourable neurological outcomes compared to those with unfavourable outcomes (standardized mean difference −1.78, 95%CI: −2.25 to −1.31; *p* < 0.001). The diagnostic accuracy was high, with pooled sensitivity and specificity of 0.63 and 0.93, respectively, and an area under the curve of 0.89 (95% CI 0.85–0.92). Subgroup and sensitivity analyses confirmed the robustness of these findings across various study populations and temporal points, with negligible evidence of publication bias. **Conclusions:** These results indicate that serum S100B is a reliable early biomarker of neurological prognosis after CA. Incorporating S100B into multimodal predictive frameworks may enhance post-resuscitation decision-making.

## 1. Introduction

Cardiac arrest (CA) remains one of the significant public health challenges worldwide. In developed countries, the incidence of CA is estimated at 53 to 90 cases per 100,000 people annually, with fewer than 12% of patients surviving to hospital discharge [1,2]. To improve patient outcomes, it is essential to recognize the problem promptly, initiate cardiopulmonary resuscitation (CPR) without delay, and provide advanced care as soon as possible after return of spontaneous circulation (ROSC) [3,4]. In spite of significant progress in resuscitation skills and critical care, the principal factor influencing long-term survival and quality of life continues to be the severity of hypoxic–ischemic brain injury incurred during the arrest [5,6]. Accurate neurological prognostication following CA is crucial for informing therapeutic choices, facilitating communication with families and caregivers, and enhancing the allocation of healthcare resources [6,7]. Standard prognostic instruments, such as neurological examination, electroencephalography (EEG), neuroimaging, and evoked potential testing, require expert interpretation and may not be readily accessible during the acute phase [5,7]. As a result, there is growing interest in circulating biomarkers of brain injury that can deliver swift, objective, and reproducible prognostic data [8,9,10].

S100B is a protein that binds to calcium and is mainly found in astrocytes and Schwann cells. Following glial injury and the disruption of the blood–brain barrier, it is released into the extracellular space and bloodstream [10,11]. At the molecular level, S100B acts as a damage-associated molecular pattern (DAMP) that connects cellular damage and neuroinflammatory signalling. Under normal circumstances, S100B has neurotrophic and neuroprotective effects, helping neurons survive, organize their cytoskeleton, and alter their connectivity through low-level autocrine and paracrine signalling. After an ischemic or hypoxic injury, however, reactive astrocytes release too much S100B, which raises extracellular levels to a point where they cause neurotoxic and proinflammatory responses.

The S100B attaches to the receptor for advanced glycation end products (RAGE) on microglia, astrocytes, and endothelial cells. This initiates signalling pathways, including NF-κB, p38 MAPK, and ERK1/2 [10,11]. Next, this activation promotes the release of proinflammatory cytokines (such as TNF-α, IL-1β, and IL-6) and reactive oxygen species, which worsen secondary neuronal injury by inducing oxidative stress, excitotoxicity, and dysfunction of the blood–brain barrier. Moreover, S100B-RAGE interactions facilitate endothelial activation, vasogenic edema, and microvascular dysfunction, thereby intensifying cerebral injury. This concentration-dependent dichotomy, which is neuroprotective at low levels and detrimental at high levels, is frequently referred to as the “double-edged sword” phenomenon of S100B signalling [10,11,12]. High levels of S100B have been consistently linked to widespread neuronal and glial cell damage and poor neurological outcomes after CA, as shown by several observational studies and meta-analyses [5,8,13,14,15,16]. Recent findings suggest that S100B functions as a significant early prognostic biomarker, especially within the first 24–48 h following CA, facilitating prompt recognition of patients at elevated risk of suboptimal neurological recovery [5,8,13,14,15,16,17]. When integrated into multimodal prognostic models that include clinical data, neuroimaging results, and other biomarkers such as neuron-specific enolase (NSE), S100B improves predictive accuracy and supports more personalized treatment decisions [6,15,16]. According to the most recent American Heart Association (AHA) guidelines, biomarkers such as S100B should be included in comprehensive prognostic algorithms to improve care after CPR and guide clinical decisions, supported by substantial evidence [1,9].

Numerous studies have investigated the correlation between S100B levels and neurological outcomes after CA; however, findings are inconsistent due to differences in sampling times, assay techniques, and outcome definitions. A thorough synthesis of the existing evidence is necessary to elucidate the prognostic significance of S100B in this context. The objective of this meta-analysis was to systematically assess the diagnostic and prognostic significance of serum S100B concentrations for predicting adverse neurological outcomes following CA, and to determine optimal sampling time points and cutoff thresholds to improve its clinical utility within multimodal prognostic frameworks.

## 2. Materials and Methods

### 2.1. Protocol, Registration, and Reporting

This systematic review and meta-analysis were designed a priori in accordance with the PRISMA 2020 statement and its PRISMA-DTA extension for diagnostic and prognostic accuracy studies (PRISMA checklist in Table S1) [18]. The study protocol will be registered in the PROSPERO database prior to data extraction (registration ID: CRD420251182587). As this research involved the secondary analysis of previously published, de-identified data and did not include any new human or animal participants, ethical approval and informed consent were not required, in accordance with institutional and international guidelines.

### 2.2. Information Sources and Search Strategy

A thorough literature search was conducted across various electronic databases, including PubMed (MEDLINE), Embase, Scopus, Web of Science, Cochrane Central Register of Controlled Trials (CENTRAL), and CINAHL, from the inception of the databases to 3 November 2025. The search strategy was devised a priori to optimize both sensitivity and specificity, utilizing a blend of controlled vocabulary terms (MeSH and Emtree) and free-text keywords pertinent to S100B, CA, and neurological outcomes. The search strategy combined controlled vocabulary and free-text terms. For the primary database query, the following keywords and their variations were applied: (“S100B” OR “S100”) AND (“cardiac arrest” OR “out-of-hospital cardiac arrest” OR “OHCA” OR “in-hospital cardiac arrest” OR “heart arrest” OR “cardiopulmonary resuscitation” OR “CPR” OR “sudden cardiac death”).

This strategy was customized for each database using syntax and field tags specific to each platform. We used Boolean operators, truncation symbols, and proximity functions when they were needed. There were no restrictions on publication date to ensure a complete search, but the analysis included only articles published in English. Table S2 in the Supplementary Materials shows the full search strategies for each database, including all keywords, MeSH/Emtree terms, Boolean operators, and syntax changes.

In addition to database searches, manual and additional searches were conducted to identify studies not listed in standard databases. These included checking the reference lists of all included articles and systematic reviews, as well as grey literature sources such as Google Scholar, preprint repositories, and trial registries (ClinicalTrials.gov and the WHO International Clinical Trials Registry Platform).

All citations found were imported into EndNote (Clarivate Analytics, Philadelphia, PA, USA) to remove duplicates. Then, they were uploaded to Rayyan (Qatar Computing Research Institute; Doha, State of Qatar) to make screening them easier without knowing who they were. Two reviewers independently evaluated titles, abstracts, and full-text articles based on established inclusion and exclusion criteria. Any differences in opinion among the reviewers were discussed and resolved with the senior author’s help. This made sure that the study selection was consistent and agreed upon.

### 2.3. Eligibility Criteria

The PECOS framework (Population, Exposure, Comparator, Outcomes, Study design) was used to define the inclusion criteria, ensuring the study was consistent and methodologically clear.

The population (P) consisted of adult patients (≥18 years) who achieved ROSC after out-of-hospital or in-hospital CA. Studies that included patients with CA due to primary neurological causes, like traumatic brain injury, subarachnoid haemorrhage, or intracerebral haemorrhage, were not included. Studies that included only children were also excluded. There were no limitations concerning the initial rhythm, aetiology, or the implementation of targeted temperature management (TTM).

The exposure (E) of interest was the serum concentration of the S100B protein measured in peripheral blood within 72 h after ROSC, regardless of the analytical platform or assay employed. When possible, the exact sampling window (≤6 h, 24 h, 48 h, or 72 h) was used to account for time-dependent changes in biomarkers.

The comparator (C) was established as the disparity in S100B levels between outcome groups, specifically patients with favourable versus unfavourable neurological outcomes, or survivors versus non-survivors at hospital discharge. For diagnostic accuracy analyses, studies that specified S100B cut-off thresholds for outcome prediction were included.

The outcomes (O) of interest were both prognostic and diagnostic. The primary outcomes comprised (1) an unfavourable neurological outcome, characterized by a Cerebral Performance Category (CPC) of 3–5 or an equivalent scale (e.g., Glasgow Outcome Scale ≤ 3, modified Rankin Scale ≥ 4) at hospital discharge or after a minimum of 3 months of follow-up; and (2) survival until hospital discharge. Secondary outcomes encompassed the area under the receiver operating characteristic curve (AUC), sensitivity, specificity, positive and negative likelihood ratios (LR+/LR−), diagnostic odds ratio (DOR), and standardized mean or log-transformed differences in S100B concentrations among outcome groups.

The study design (S) criteria included original observational studies, whether prospective or retrospective cohorts, and observational arms of randomized controlled trials that provided extractable quantitative data for at least one predetermined outcome. Case series with fewer than ten participants, conference abstracts lacking complete data, narrative reviews, editorials, and animal studies were omitted. There were no limits on language or publication date, and studies that were not in English were translated when needed.

Studies were excluded if they failed to adhere to the predetermined PECOS framework or if their design, population, or reporting obstructed reliable data extraction. Specifically, studies were deemed ineligible if they involved paediatric patients under 18 years of age or comprised mixed adult-paediatric cohorts in which adult data could not be independently analysed. Reports examining CA of predominantly neurological or traumatic aetiology, including traumatic brain injury, intracerebral haemorrhage, or subarachnoid haemorrhage, were excluded to prevent biological confounding associated with extracerebral S100B release. Non-original research, including review articles, editorials, conference abstracts, animal experiments, and case series with fewer than 10 participants, was excluded. In cases where several publications discussed overlapping cohorts, only the most detailed or the latest report was kept. Furthermore, studies were omitted if S100B levels explicitly influenced clinical decisions concerning the cessation of life-sustaining therapy, or if significant methodological deficiencies—such as unverified assays, unblinded outcome assessment, or inconsistent reporting of measurement units—were detected during data evaluation.

The search was limited to studies published in English to guarantee uniformity in methodological and terminological interpretation. These criteria were uniformly applied to ensure methodological rigor, mitigate potential sources of bias, and maintain the validity of the aggregated estimates.

### 2.4. Data Extraction

Data extraction was performed using a standardized, pre-designed form created for this review, in alignment with PRISMA 2020 [18]. The extraction template was tested on a small group of eligible studies to ensure that the data collection was clear, consistent, and reproducible. Two reviewers independently extracted all pertinent information from each included study. The extracted data encompassed study characteristics (first author, publication year, country, and study design), population specifics (sample size, mean or median age, sex distribution, type of CA (OHCA or IHCA), and implementation of TTM), exposure information (timing of S100B sampling post-ROSC, biological matrix, assay type and analytical platform, and reported cutoff values), and outcome metrics (neurological outcome assessed via the Cerebral Performance Category (CPC) [19], Glasgow Outcome Scale (GOS) [20], modified Rankin Scale (mRS) [21], and survival to hospital discharge). Any differences in opinion between the two reviewers were discussed and resolved by consensus. If any remaining issues remained to be settled, the senior author addressed them. When essential data (e.g., standard deviations, cut-off values, or outcome definitions) were missing or unclear, the corresponding authors of the included studies were contacted. We put all the data we obtained into a secure electronic database (Microsoft Excel, Microsoft Corp., Redmond, WA, USA) and checked it for accuracy and consistency. The completed dataset was subsequently imported into R (version 4.3.0; R Core Team, Vienna, Austria) for meta-analytic synthesis.

### 2.5. Risk of Bias Assessment

The Newcastle-Ottawa Scale (NOS) was used to rate the methodological quality and risk of bias of the included observational studies [22]. The NOS is made for cohort and case–control studies. This tool assesses the quality of a study in three areas: how well the study groups were selected, how similar the cohorts are, and how well the outcomes were measured.

Two reviewers evaluated each study separately and used the NOS criteria that had already been set. Consensus discussions were used to resolve any scoring differences. If no agreement could be reached, the senior author made the final decision to ensure the methods were consistent and clear.

In cohort studies, the maximum possible points were 9:4 for selection, 2 for comparability, and 3 for outcome assessment. Studies that scored 7–9 points were considered to have a low risk of bias. Studies that scored 5–6 points were considered to have a moderate risk of bias, and those that scored four or less were considered to have a high risk of bias.

When assessing selection bias, the focus was on how well the cohort represented the population, the sample size, and the measurement of exposure (i.e., S100B levels). The comparability domain assessed the extent to which studies mitigated potential confounders, including age, initial cardiac rhythm, TTM implementation, and timing of biomarker sampling. The outcome domain focused on the validity and reliability of neurological and survival endpoints, the blinding of outcome assessors, and the adequacy of the follow-up duration and completeness.

### 2.6. Quality Assessment

We used the GRADE (Grading of Recommendations, Assessment, Development and Evaluation) framework to assess the certainty of the evidence across all outcomes [23]. This was done in accordance with the rules for studies on prognostic factors and diagnostic accuracy. The GRADE method allows you to assess how confident you are in pooled estimates by considering key factors that may affect the reliability and generalizability of the results.

Two reviewers used the GRADE criteria to rate each primary and secondary outcome separately. Disagreements were settled by consensus, with the senior author stepping in to mediate when needed. Cohort studies were initially given high-quality ratings, but these ratings were later changed in accordance with criteria that had already been set.

Five areas were looked at for downgrading: risk of bias (methodological problems with the studies that were included), inconsistency (differences in effect estimates between studies), indirectness (how well the evidence applies to the target population or outcomes), imprecision (how wide the confidence intervals are and how big the sample size is), and publication bias (asymmetry in funnel plots or selective reporting). Evidence could be upgraded if studies showed a significant or consistent effect size, a clear dose–response relationship, or if all possible residual confounding would lower the observed association instead of raising it. The overall assessment of these areas resulted in a rating of high, moderate, low, or very low for the certainty of the evidence for each outcome.

### 2.7. Data Synthesis and Meta-Analysis

We used standardized mean differences (SMDs) with 95% confidence intervals (CIs) to combine continuous outcomes, such as serum S100B concentrations. Hedges’ g correction for small-sample bias was used to do this. When studies presented outcomes as medians with ranges or interquartile ranges, the corresponding means and standard deviations were estimated using the methodology proposed by Hozo and colleagues [24]. Cochran’s Q test and the I^2^ statistic were used to assess between-study heterogeneity. The I^2^ statistic shows the proportion of total variation due to heterogeneity rather than chance [25].

A random-effects model (DerSimonian-Laird method) was employed to address expected inter-study variability. The Cochran’s Q test was used to measure between-study heterogeneity, and the I^2^ statistic was used to quantify it. Values of 25%, 50%, and 75% showed low, moderate, and high heterogeneity, respectively.

Subgroup analyses were performed based on the aetiology of CA (out-of-hospital versus in-hospital), geographic region (Asia versus Europe/North America), and the timing of biomarker assessment (baseline, 24 h, 48 h, 72 h, and 96 h post-ROSC).

Sensitivity analyses using the leave-one-out (LOO) approach were performed to assess the stability of the pooled estimates by sequentially excluding each individual study.

Funnel plots were used to visually assess publication bias, and the trim-and-fill method was used to assess publication bias statistically. The symmetry of the funnel plots and the absence of imputed studies were considered indicators of minimal publication bias.

To assess the diagnostic accuracy of S100B in predicting the primary outcome, neurological status at six months post-CA, pooled sensitivity, specificity, and their respective 95% confidence intervals were computed at various time points (baseline, 24 h, 48 h, and 72 h post-ROSC). Summary receiver operating characteristic (SROC) curves were generated, and the area under the curve (AUC) was calculated to measure the overall discriminative efficacy of S100B. The Q* index was created to show the best balance between sensitivity and specificity.

We performed all statistical analyses using R software (version 4.3.0; R Foundation for Statistical Computing, Vienna, Austria) and Stata (version 18.0; StataCorp, College Station, TX, USA). In R, the following packages were employed: meta (for random-effects meta-analysis and forest plots), metafor (for effect size estimation and heterogeneity testing), mada (for diagnostic accuracy and SROC analyses), dmetar (for influence diagnostics and publication bias assessment), and ggplot2 (for data visualization). For all analyses, *p* < 0.05 was the cutoff for statistical significance.

## 3. Results

### 3.1. Study Selection

**Figure 1:**
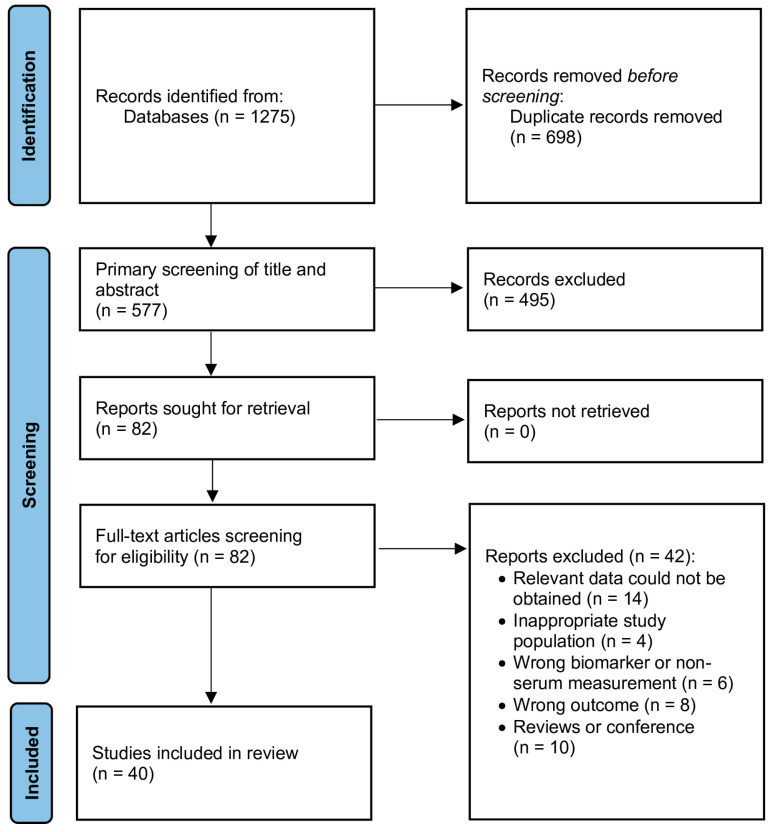
PRISMA 2020 flow diagram of the study selection process.

Based on the information depicted in Figure 1, a total of 1275 publications were identified through database searches. After removal of duplicate records, 577 unique entries were screened for eligibility. After reviewing the titles and abstracts, 495 studies were excluded for not meeting the inclusion criteria. After that, 82 were assessed for eligibility through a detailed review. Of those, 40 met all the criteria set and were used in the quantitative synthesis [5,13,14,15,16,17,26,27,28,29,30,31,32,33,34,35,36,37,38,39,40,41,42,43,44,45,46,47,48,49,50,51,52,53,54,55,56,57,58,59,60,61,62,63,64,65,66]. All studies included were observational in design and published between 2005 and 2025.

### 3.2. Study Characteristics

**Figure 2:**
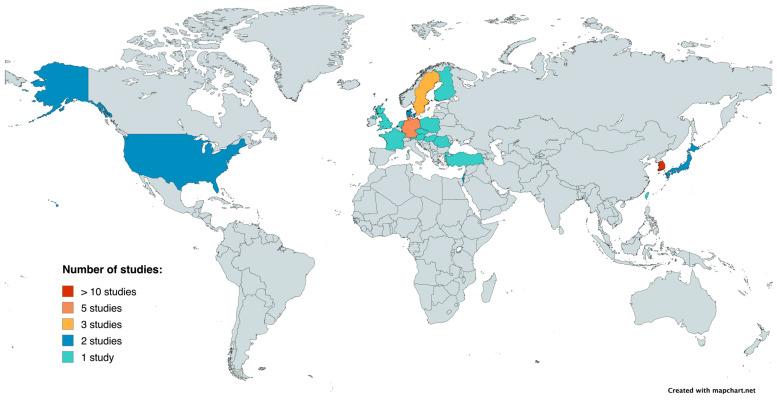
Geographic distribution of the included studies.

Baseline characteristics of the study populations across the included trials are presented in Table S3. The studies included came from 15 countries in Europe, Asia, and the United States, showing a wide range of healthcare and geographic diversity. Cohorts were conducted in Europe in Germany, France, Sweden, Denmark, Poland, the Czech Republic, Romania, Austria, Finland, Luxembourg, the Netherlands, the United Kingdom, Türkiye, and Israel. Asian cohorts were conducted in Korea, Japan, and Taiwan. The United States conducted two studies [5,31]. The geographical distribution of the studies included in the meta-analysis is illustrated in Figure 2.

The proportion of male patients ranged from 60% to 80% across cohorts. Most participants were middle-aged or older adults, with a mean or median age of 48 to 72 years. Studies included mostly OHCA cases (60–100%), with the remainder comprising a mix of OHCA and in-hospital populations. Witnessed arrests accounted for 50–95% of the total, and bystander-initiated CPR accounted for 20–90%. Approximately 50–65% of patients had a shockable primary rhythm, which is in line with what is seen in modern international CA registries.

### 3.3. Primary Outcome: Neurological Status at 6-Month Follow-Up

**Figure 3:**
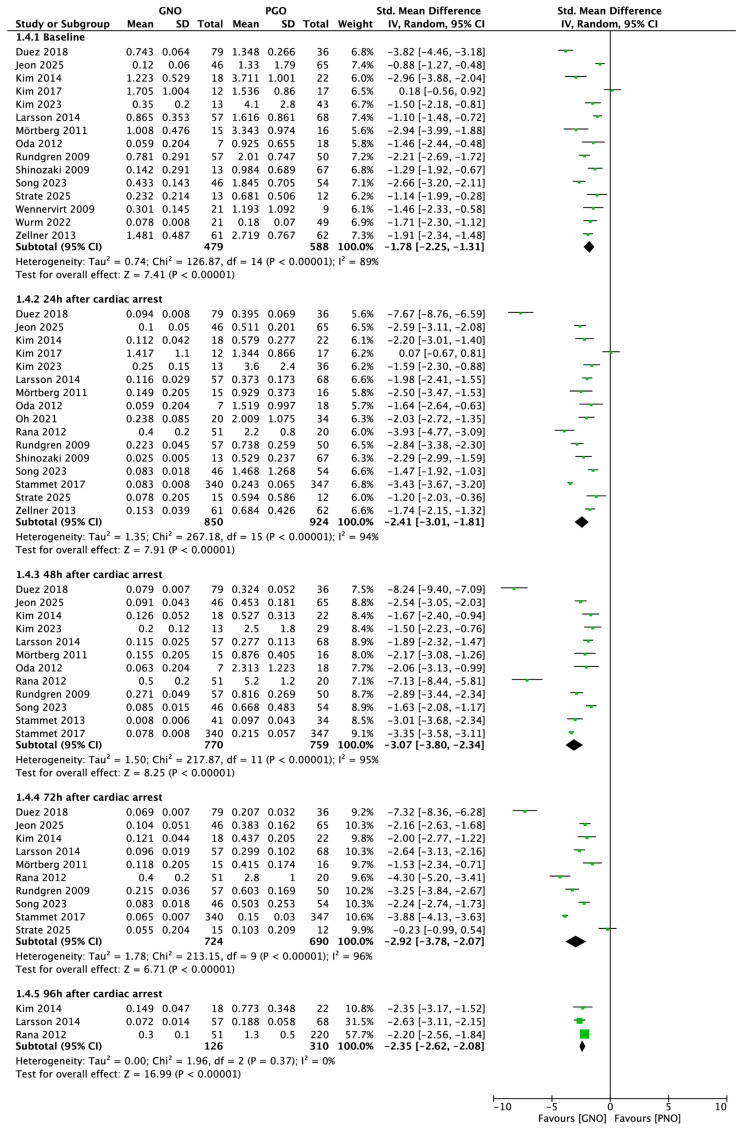
Forest plot presenting standardized mean differences (SMD) in S100B levels between patients with good and poor 6-month neurological outcomes at baseline and at 24, 48, 72, and 96 h after cardiac arrest.

The pooled analysis indicated that serum S100B concentrations assessed post-ROSC were markedly lower in patients with favourable neurological outcomes at six months compared to those with unfavourable outcomes (0.717 ± 0.557 vs. 1.805 ± 1.547 µg/L; SMD = −1.78; 95% CI, −2.25 to −1.31; *p* < 0.001; Figure 3).

Subgroup analyses validated the robustness of this association among patients with OHCA (SMD = −1.09; 95% CI, −1.49 to −0.70; *p* < 0.001), as well as within both Asian (SMD = −1.43; 95% CI, −1.97 to −0.90; *p* < 0.001) and European/North American (SMD = −0.91; 95% CI, −1.29 to −0.54; *p* < 0.001; Table S4) cohorts.

**Figure 4:**
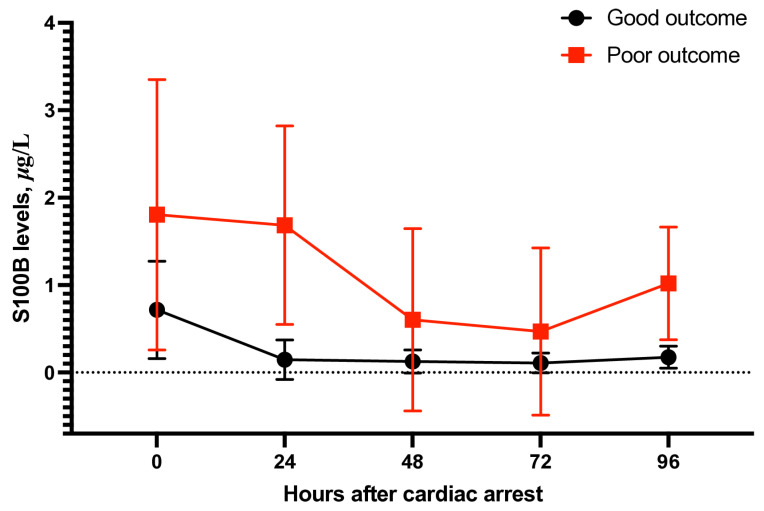
S100B levels (mean ± SD) at 0, 24, 48, 72, and 96 h after cardiac arrest in patients with good and poor 6-month neurological outcomes.

Repeated measurements showed that patients with good neurological recovery always had lower S100B levels. On day 1 post-ROSC, the mean concentrations were 0.146 ± 0.225 µg/L for patients with favourable outcomes and 0.734 ± 1.010 µg/L for those with unfavourable outcomes (SMD = −2.41; 95% CI, −3.01 to −1.81; *p* < 0.001; Figure 4). The difference remained significant on day 2 (0.109 ± 0.113 vs. 0.341 ± 0.495 µg/L; SMD = −3.07; 95% CI, −3.80 to −2.34; *p* < 0.001) and persisted through days 3 and 4 across all subgroups (*p* < 0.001).

### 3.4. Secondary Neurological Outcomes

At every time point examined, serum S100B levels were consistently and significantly lower in patients with good neurological outcomes than in those with poor outcomes at hospital discharge (all *p* < 0.001; Figures S1 and S2). The standardized mean differences ranged from −1.41 to −3.54, indicating a large effect size. This pattern persisted across OHCA, Asian, and European/North American subgroups, notwithstanding significant heterogeneity (I^2^ 85–97%; Table S5).

In general, S100B levels decreased over time in survivors with good neurological function, but remained high in those with poor recovery. This further supports its usefulness as a predictor at hospital discharge.

After 3 months, combined data from 2 studies indicated that S100B concentrations remained significantly lower in patients with positive neurological recovery (SMD = −3.30; 95% CI, −4.08 to −2.51; *p* < 0.001; I^2^ = 80%; Figures S3 and S4). Results were consistent across all time points (24–72 h) and subgroups, with substantial and uniform effect sizes (SMD range: −2.94 to −3.79; all *p* < 0.001; Table S6).

### 3.5. Exploratory Analysis: S100B as a Prognostic Marker for Survival

Eight studies assessed serum S100B concentrations as prognostic indicators of survival post-ROSC. The pooled data showed that survivors had much lower S100B levels than non-survivors (SMD = −1.03; 95% CI, −1.83 to −0.22; *p* = 0.01; Figure 4). In the OHCA subgroup, survivors exhibited reduced levels (SMD = −0.93; 95% CI, −1.83 to −0.02; *p* = 0.04), and a comparable trend was observed in European/North American cohorts (SMD = −1.27; 95% CI, −2.31 to −0.22; *p* = 0.02). No statistically significant association was detected among Asian populations (SMD = −0.30; 95% CI, −0.63 to 0.03; *p* = 0.07; Table S7).

At 24 h post-ROSC, S100B concentrations were significantly lower in survivors (0.101 ± 0.408 µg/L) compared to non-survivors (0.814 ± 1.205 µg/L; SMD = −1.61; 95% CI, −1.89 to −1.34; *p* < 0.001; Figure S5). The OHCA subgroup showed similar results (SMD = −1.72; 95% CI, −2.03 to −1.41; *p* < 0.001).

At 48 and 72 h, survivors consistently demonstrated significantly diminished S100B concentrations (SMD = −1.58 and −2.68, respectively; all *p* < 0.001; Figure S6), with the most pronounced effect noted at 72 h (SMD = −3.64; 95% CI, −3.64 to −1.78; *p* < 0.001).

Only one study (Petermichl et al. [47]) provided data at 96 h, indicating a comparable trend (SMD = −1.64; 95% CI, −2.24 to −1.04; *p* < 0.001). 

### 3.6. Diagnostic Accuracy of S100B

A summary receiver operating characteristic meta-analysis was conducted to evaluate the diagnostic efficacy of S100B in predicting unfavourable neurological outcomes. The pooled sensitivity and specificity right after ROSC were 0.63 (95% CI: 0.53–0.72) and 0.93 (95% CI: 0.86–0.97), respectively (Table S8). The summary AUC was 0.89 (95% CI: 0.85–0.92), indicating that the test’s overall accuracy was very high. At 24 h, the pooled sensitivity was 0.50 (95% CI: 0.34–0.65) and the specificity was 0.91 (95% CI: 0.83–0.96), which means the test was not very good at diagnosing (AUC = 0.57; 95% CI: 0.49–0.65).

After 48 h, diagnostic performance improved, with a pooled sensitivity of 0.48 (95% CI: 0.36–0.60), a specificity of 0.96 (95% CI: 0.91–0.99), and an AUC of 0.81 (95% CI: 0.75–0.87). The Q* index, which shows the best balance between sensitivity and specificity, was 0.74 (95% CI: 0.68–0.80).

The pooled sensitivity fell to 0.35 (95% CI: 0.24–0.48) after 72 h, but the specificity stayed very high at 0.97 (95% CI: 0.91–0.99). The AUC was 0.74 (95% CI: 0.67–0.81) and Q* was 0.69 (95% CI: 0.61–0.76).

These results show that early measurements (within 24 h) are moderately accurate for predicting outcomes, but S100B peaks between 48 and 72 h after ROSC are better at distinguishing between groups, with both specificity and overall accuracy highest.

### 3.7. Bias in Publication and Sensitivity Analysis

The leave-one-out sensitivity analysis validated the robustness of the aggregated estimates. Sequential exclusion of individual studies did not significantly modify the overall effect size, which varied from Hedges’ g = −1.62 (95% CI: −2.06 to −1.19) to Hedges’ g = −1.91 (95% CI: −2.36 to −1.46; Figure S7), with all results retaining statistical significance (*p* < 0.001). No single study unduly influenced the meta-analytic model, thereby affirming the stability of the results.

A visual inspection of the funnel plot showed that the studies were evenly distributed around the pooled effect estimate, suggesting slight publication bias (Figure S8). The trim-and-fill method, which did not add any missing studies, confirmed this by giving the same adjusted pooled effect (Hedges’ g = −1.78; 95% CI: −2.28 to −1.28). These analyses collectively demonstrate a minimal risk of bias and a high level of methodological robustness in the synthesized findings.

### 3.8. Certainty of Evidence

Table S9 presents the GRADE assessment of the certainty of the evidence for S100B measured at different times after ROSC in predicting neurological outcome at 6 months. The overall level of certainty in the evidence was moderate for biomarker measurements taken between baseline and 72 h, and very low for measurements taken at 96 h because there was not enough data.

At all early time points, the effect direction was highly consistent, with higher S100B levels strongly associated with poor outcomes. At baseline (0–6 h), the evidence was moderately specific, with a large pooled effect size (SMD of about −1.8 to −2.2), but there was substantial heterogeneity. After 24 h, the evidence was still moderate, but there was a strong and precise effect (SMD −2.41; 95% CI −3.01 to −1.81). At 48 h, the highest level of certainty and effect size was seen, with S100B showing the most significant and most stable prognostic effect (SMD −3.07; 95% CI −3.80 to −2.34). These findings meant that it should be moved up to the moderate category.

At 72 h, the strength of the association remained strong (SMD −2.76; 95% CI −3.71 to −1.82), but the studies remained heterogeneous, so the overall certainty rating was moderate. Measurements at 96 h, however, were corroborated by only one or two studies, indicating significant imprecision and severe inconsistency, resulting in a classification of very low certainty.

These findings collectively suggest that S100B has significant prognostic value within the first 72 h post-ROSC, with the 48 h measurement identified as the most reliable and distinguishing time point for predicting long-term neurological outcomes.

## 4. Discussion

CA remains one of the most challenging conditions in critical care, frequently leading to cerebral hypoxia and subsequent neurological injury. Accurate and early prediction of neurological outcomes after ROSC is essential for guiding treatment decisions and supporting family counselling. This meta-analysis demonstrates that serum S100B levels measured after ROSC are significantly lower in patients with favorable neurological recovery compared to those with poor outcomes.

The combined standardized mean difference of −1.78 (95% CI: −2.25 to −1.31; *p* < 0.001) indicates a strong, consistent effect size. Diagnostic accuracy analyses further validated exceptional discriminatory performance, exhibiting pooled sensitivity and specificity of 0.63 and 0.93, respectively, alongside an overall AUC of 0.89 (95% CI 0.85–0.92). Subgroup analyses corroborated these findings within OHCA, Asian, and European/North American cohorts. Sensitivity analyses and funnel-plot symmetry testing demonstrated stable estimates and negligible publication bias.

### 4.1. Mechanistic Interpretation

S100B is a calcium-binding (Ca^2+^) protein from the S100 family. Astrocytes in the central nervous system produce and release it following hypoxic–ischemic injury [10,61]. After a CA, a rapid rise in serum S100B levels indicates that many astrocytes are activated and injured, the blood–brain barrier is disrupted, and the glymphatic system is activated, facilitating the protein’s rapid entry into the peripheral circulation [17,62].

S100B is not only a biomarker but also an active signalling molecule of the damage-associated molecular pattern (DAMP) type. When S100B is released into the extracellular space, it binds to the receptor for advanced glycation end-products (RAGE) on the surface of astrocytes, microglia, and neurons. This starts a chain reaction of intracellular signalling pathways, including the activation of NF-κB, MAPK, and PI3K/Akt, as well as the production of proinflammatory cytokines and chemokines [61,63,64,65]. These effects depend on concentration: S100B may protect neurons and promote growth at low concentrations, but at high (micromolar) levels, it promotes a proinflammatory astrocytic phenotype, increases microglial migration, induces oxidative stress, and leads to neuronal death [63,64].

In the setting of CA, S100B demonstrates a distinct biphasic kinetic profile. In serum, a rapid, early peak is observed within the first few hours after ROSC. This phenomenon occurs because astrocytes suddenly release the substance, which then quickly passes through the blood–brain barrier and the glymphatic pathways [17,62,66]. Then, there is a quick drop, linked to renal clearance and reduced efflux from the central nervous system as barrier integrity and glymphatic flow worsen [17,62]. In cerebrospinal fluid, S100B levels, however, remain elevated for an extended period, suggesting that astrocytic injury persists [17].

### 4.2. Clinical Implications

Numerous prospective and multicentre studies have confirmed the prognostic relevance of S100B as an early biomarker after CA. Deye et al. showed that measuring S100B at admission provided the best early indication of a severe neurological outcome at 3 months, with an AUC of 0.83. Adding S100B to clinical models improved net reclassification indices, indicating that it adds value to early prognostication frameworks [13].

Mörtberg et al. found that S100B levels measured 24 h post-ROSC were predictive of unfavourable neurological outcomes, with high sensitivity and specificity [42]. Furthermore, S100B demonstrated superior performance compared to other biomarkers, including NSE and glial fibrillary acidic protein (GFAP), in patients undergoing TTM [54]. Hoiland et al. conducted a thorough meta-analysis and found that S100B had a pooled AUC of 0.85 (95% CI, 0.76–0.92) for predicting poor neurological outcomes, with the best results coming in the early post-arrest period [5]. These findings are consistent with other systematic reviews and meta-analyses, which have indicated that S100B is a particular marker for early identification of poor prognosis, particularly when used in conjunction with other clinical and biochemical parameters [5,12].

The AHA, the European Resuscitation Council, and the European Society of Intensive Care Medicine all recommend using S100B as an additional biomarker within a multimodal prognostic framework for post-CA care. They stress that S100B should not be used alone but rather as part of a whole approach to neuroprognostication [67,68]. These recommendations are based on substantial evidence indicating that serial S100B measurements within the first 72 h post-CA yield significant prognostic information that can guide clinical decision-making and family counselling. Targeted temperature management might also affect the temporal kinetics of S100B. Experimental and clinical evidence indicates that hypothermia (32–36 °C) may mitigate secondary astroglial injury, stabilize the blood–brain barrier, and diminish neuroinflammatory signaling, potentially leading to reduced peak S100B concentrations and delayed release in comparison to normothermia. So, differences in TTM protocols between studies may help explain why the reported S100B kinetics and cut-off values are different. From a clinical standpoint, S100B measurements must be interpreted in conjunction with the employed temperature strategy and the timing of sampling, rather than as absolute values detached from post-resuscitation care.

### 4.3. Strengths and Future Trajectories

This meta-analysis is the most thorough synthesis to date of longitudinal S100B measurements collected at different times after ROSC across patient groups. It includes both prognostic and diagnostic performance outcomes. Its main strengths are a consistent six-month neurological endpoint, strong performance metrics such as the AUC and Q* indices, and extensive sensitivity analyses that demonstrate that pooled estimates are stable and can be replicated. This analysis synthesizes data from studies with diverse designs and populations, offering a comprehensive overview of S100B kinetics and its prognostic significance following CA.

Future multicentre studies should focus on establishing standardized pre-analytical and analytical protocols, particularly for calibration and inter-assay harmonization across electrochemiluminescence (ECLIA) platforms. Setting clinically useful S100B cut-off values with a set specificity (false-positive rate = 0) and testing them in outside groups will be crucial for putting them into practice. Additionally, composite biomarker panels integrating S100B with NSE, GFAP, or NfL should be investigated, with a focus on temporal biomarker dynamics rather than single-time-point assessments. Compartment-specific studies that combine cerebrospinal fluid-serum kinetics with advanced non-invasive tests of blood–brain barrier integrity help us understand how S100B works better and make it a more critical part of multimodal prognostication.

### 4.4. Limitations

Even though the inclusion criteria were strict and the analytical methods were thorough, there are still limitations to consider. Significant heterogeneity among studies (I^2^ = 85–97%) probably shows that the studies had different designs, patient selection, and post-CA management strategies. Additionally, the lack of a universally standardized analytical reference range for S100B, coupled with inter-assay variability and variations in sampling time points, significantly constrains the immediate clinical utility of absolute cut-off thresholds. Therefore, S100B concentrations must be interpreted with caution and primarily within a multimodal neuroprognostication framework rather than as independent decision thresholds. The variations in S100B concentrations across studies can be attributed to differences in sampling time (ranging from immediately post-ROSC to 96 h), assay methods (ELISA, ECLIA, or multiplex Luminex), and calibration standards. The lack of a universally standardized analytical reference range or external quality control program hinders direct interlaboratory comparison.

The majority of the included studies were observational, which may have resulted in residual confounding due to patient demographics, comorbidities, or in-hospital management. Extracerebral S100B sources, such as adipose tissue, melanocytes, and myocardium, may affect baseline variability, especially in cases of trauma, shock, or reperfusion injury. Importantly, extracerebral sources of S100B, including adipose tissue, skeletal muscle, and myocardium, may contribute to elevated serum concentrations in the early post-resuscitation phase (0–6 h). This effect may be particularly relevant in traumatic cardiac arrest, prolonged low-flow states, reperfusion injury, or multi-organ failure, where systemic tissue damage and endothelial dysfunction can result in non-neurological S100B release. Consequently, baseline S100B measurements may be less specific for isolated hypoxic–ischemic brain injury and should be interpreted with caution when used for early neuroprognostication. Renal or hepatic dysfunction, which affects S100B clearance, was inconsistently reported and may introduce systematic bias. Variations in TTM; 32–36 °C, initiation timing, sedation protocols, seizure management, and hemodynamic optimization constitute additional unquantified confounders.

Ethical and cultural disparities in the withdrawal of life-sustaining therapy (WLST) persist as a significant constraint. Differences in laws and medical practices between regions, such as the higher rate of early WLST in European groups compared to Asian groups, may make rates of bad outcomes seem higher than they really are. The absence of clinician blinding to biomarker results in specific studies engenders a potential self-fulfilling prophecy bias. Funnel-plot symmetry and trim-and-fill analyses did not reveal significant publication bias; however, selective reporting cannot be dismissed.

Finally, there is insufficient information on long-term functional and cognitive outcomes beyond six months to determine how long S100B’s prognostic value persists. Future prospective multicentre trials with standardized sampling intervals, harmonized analytical platforms, and unified definitions of neurological recovery are crucial for establishing universal cut-off thresholds, validating biomarker kinetics across diverse populations, and improving external generalizability.

## 5. Conclusions

This meta-analysis provides strong evidence that serum S100B serves as an early, reliable, and biologically plausible biomarker for hypoxic–ischemic brain injury following CA. Lower levels of S100B within the first 24 h after return of spontaneous circulation are strongly associated with favourable neurological outcomes at six months. Importantly, the prognostic significance of S100B must be interpreted in the context of potential bias arising from the lack of clinician blinding to biomarker results when making withdrawal of life-sustaining therapy decisions. This may introduce a self-fulfilling prophecy bias, particularly in observational studies. Consequently, S100B should not be used in isolation to guide WLST decisions but rather integrated into a multimodal neuroprognostication framework in accordance with current international guidelines.

## Data Availability

The data supporting the findings of this study are available from the corresponding author upon reasonable request from corresponding author (L.S.). All extracted datasets, search strategies, and analytical codes used in the current study can be shared for academic and non-commercial purposes upon a justified request.

## Supplementary Materials

The following supporting information can be downloaded at: https://www.mdpi.com/article/10.3390/jcm15010238/s1, Table S1: PRISMA checklist; Table S2: Search strategy; Table S3: Baseline characteristics of included trials; Table S4: Summary of S100B concentrations and standardized mean differences between patients with good and poor 6-month neurological outcomes at baseline and 24, 48, 72, and 96 h after cardiac arrest; Table S5: Summary of S100B concentrations and standardized mean differences between patients with good and poor hospital discharge neurological outcomes at baseline and 24, 48, 72, and 96 h after cardiac arrest; Table S6: Summary of S100B concentrations and standardized mean differences between patients with good and poor 3-month neurological outcomes at baseline and 24, 48, 72, and 96 h after cardiac arrest; Table S7: Summary of S100B concentrations and standardized mean differences between survivors and non-survivors to hospital discharge at 0, 24, 48, 72, and 96 h after cardiac arrest; Table S8: Diagnostic Performance of S100B at Different Time Points After Cardiac Arrest; Table S9: S100B measured at different time points after ROSC (Outcome: Neurological outcome at 6 months); Figure S1: Forest plot presenting standardized mean differences in S100B levels between patients with good and poor hospital discharge neurological outcomes at baseline and at 24, 48, 72, and 96 h after cardiac arrest; Figure S2: S100B levels (mean ± SD) at 0, 24, 48, 72, and 96 h after cardiac arrest in patients with good and poor hospital discharge neurological outcomes; Figure S3: Forest plot presenting standardized mean differences in S100B levels between patients with good and poor 3-month neurological outcomes at baseline and at 24, 48, 72, and 96 h after cardiac arrest; Figure S4: S100B levels (mean ± SD) at 0, 24, 48, 72, and 96 h after cardiac arrest in patients with good and poor 3-month neurological outcomes; Figure S5: Forest plot presenting standardized mean differences in S100B levels between patients who survived vs. non-survived to hospital discharge at baseline and at 24, 48, 72, and 96 h after cardiac arrest; Figure S6: S100B levels (mean ± SD) at 0, 24, 48, 72, and 96 h after cardiac arrest in patients who survived vs. non-survived to hospital discharge; Figure S7: Leave-one-out sensitivity analysis for the primary outcome, showing the influence of each study on the pooled effect size (Hedges’s g) for differences in S100B levels between outcome groups; Figure S8: Funnel plot for publication bias assessment in the primary outcome.

## Abbreviations

The following abbreviations are used in this manuscript:

AHA	American Heart Association
AUC	Area Under the Curve
CA	Cardiac Arrest
CI	Confidence Interval
CPC	Cerebral Performance Category
CPR	Cardiopulmonary Resuscitation
DAMP	Damage-Associated Molecular Pattern
DOR	Diagnostic Odds Ratio
ECLIA	Electrochemiluminescence Immunoassay
EEG	Electroencephalography
ELISA	Enzyme-Linked Immunosorbent Assay
ERK	Extracellular Signal-Regulated Kinase
GOS	Glasgow Outcome Scale
GFAP	Glial Fibrillary Acidic Protein
GRADE	Grading of Recommendations, Assessment, Development and Evaluation
IHCA	In-Hospital Cardiac Arrest
IL	Interleukin
LR+/LR−	Positive/Negative Likelihood Ratio
MAPK	Mitogen-Activated Protein Kinase
MeSH	Medical Subject Headings
mRS	Modified Rankin Scale
NF-κB	Nuclear Factor kappa-light-chain-enhancer of activated B cells
NfL	Neurofilament Light Chain
NS	Not specified
NSE	Neuron-Specific Enolase
NOS	Newcastle–Ottawa Scale
OHCA	Out-of-Hospital Cardiac Arrest
PRISMA	Preferred Reporting Items for Systematic Reviews and Meta-Analyses
PROSPERO	International Prospective Register of Systematic Reviews
RAGE	Receptor for Advanced Glycation End Products
ROSC	Return of Spontaneous Circulation
S100B	S100 Calcium-Binding Protein B
SHD	Survival to Hospital Discharge
SMD	Standardized Mean Difference
SROC	Summary Receiver Operating Characteristic
TNF-α	Tumor Necrosis Factor alpha
TTM	Targeted Temperature Management
