# Supplementary material for: Prognostic Value of Serum S100B Protein for Neurological Outcomes After Cardiac Arrest: A Systematic Review and Meta-Analysis
Source: J Clin Med. 2025 Dec 28;15(1):238. doi: 10.3390/jcm15010238 (PMC12786873; doi:10.3390/jcm15010238)
Supplement: Supplementary file 1 [file jcm-15-00238-s001.zip › jcm-4034895-supplementary.pdf]

PROGNOSTIC VALUE OF SERUM S100B PROTEIN FOR NEUROLOGICAL OUTCOME AFTER CARDIAC ARREST: A SYSTEMATIC REVIEW AND META-ANALYSIS

Supplementary Digital File

CONTENT:

Table S1. PRISMA checklist 3

Table S2. Search strategy........ 5

Table S3. Baseline characteristics of included trials. 10

Table S4. Summary of S100B concentrations and standardized mean differences between patients with good and poor 6-month neurological outcomes at baseline and 24, 48, 72, and 96 hours after cardiac arrest. 16

***Table S5.* Summary of S100B concentrations and standardized mean differences between patients with good and poor hospital discharge neurological outcomes at baseline and 24, 48, 72, and 96 hours after cardiac arrest. 17**

Table S6. Summary of S100B concentrations and standardized mean differences between patients with good and poor 3-month neurological outcomes at baseline and 24, 48, 72, and 96 hours after cardiac arrest. 18

Table S7. Summary of S100B concentrations and standardized mean differences between survivors and non-survivors to hospital discharge at 0, 24, 48, 72, and 96 hours after cardiac arrest. 19

Table S8. Diagnostic Performance of S100B at Different Time Points After Cardiac Arrest. 20

Table S9. S100B measured at different time points after ROSC (Outcome: Neurological outcome at 6 months)........ 21

Figure S1. Forest plot presenting standardized mean differences (SMD) in S100B levels between patients with good and poor hospital discharge neurological outcomes at baseline and at 24, 48, 72, and 96 hours after cardiac arrest. 22

Figure S2. S100B levels (mean ± SD) at 0, 24, 48, 72, and 96 hours after cardiac arrest in patients with good and poor hospital discharge neurological outcomes........ 23

Figure S3. Forest plot presenting standardized mean differences in S100B levels between patients with good and poor 3-month neurological outcomes at baseline and at 24, 48, 72, and 96 hours after cardiac arrest. **23**

Figure S4. S100B levels (mean ± SD) at 0, 24, 48, 72, and 96 hours after cardiac arrest in patients with good and poor 3-month neurological outcomes. **24**

Figure S5. Forest plot presenting standardized mean differences in S100B levels between patients who survived vs. non-survived to hospital discharge at baseline and at 24, 48, 72, and 96 hours after cardiac arrest. **25**

Figure S6. S100B levels (mean ± SD) at 0, 24, 48, 72, and 96 hours after cardiac arrest in patients who survived vs. non-survived to hospital discharge. **26**

Figure S7. Leave-one-out sensitivity analysis for the primary outcome, showing the influence of each study on the pooled effect size (Hedges's g) for differences in S100B levels between outcome groups. **27**

Figure S8. Funnel plot for publication bias assessment in the primary outcome........................ **28**

Table S1. PRISMA checklist

Section and Topic	Item #	Checklist item	Location where item is reported
**TITLE**			
Title	1	Identify the report as a systematic review.	1
**ABSTRACT**			
Abstract	2	See the PRISMA 2020 for Abstracts checklist.	1,2
**INTRODUCTION**			
Rationale	3	Describe the rationale for the review in the context of existing knowledge.	2
Objectives	4	Provide an explicit statement of the objective(s) or question(s) the review addresses.	3
**METHODS**			
Eligibility criteria	5	Specify the inclusion and exclusion criteria for the review and how studies were grouped for the syntheses.	4,5
Information sources	6	Specify all databases, registers, websites, organisations, reference lists and other sources searched or consulted to identify studies. Specify the date when each source was last searched or consulted.	3
Search strategy	7	Present the full search strategies for all databases, registers and websites, including any filters and limits used.	3,4
Selection process	8	Specify the methods used to decide whether a study met the inclusion criteria of the review, including how many reviewers screened each record and each report retrieved, whether they worked independently, and if applicable, details of automation tools used in the process.	5
Data collection process	9	Specify the methods used to collect data from reports, including how many reviewers collected data from each report, whether they worked independently, any processes for obtaining or confirming data from study investigators, and if applicable, details of automation tools used in the process.	5
Data items	10a	List and define all outcomes for which data were sought. Specify whether all results that were compatible with each outcome domain in each study were sought (e.g. for all measures, time points, analyses), and if not, the methods used to decide which results to collect.	5
	10b	List and define all other variables for which data were sought (e.g. participant and intervention characteristics, funding sources). Describe any assumptions made about any missing or unclear information.	5
Study risk of bias assessment	11	Specify the methods used to assess risk of bias in the included studies, including details of the tool(s) used, how many reviewers assessed each study and whether they worked independently, and if applicable, details of automation tools used in the process.	5
Effect measures	12	Specify for each outcome the effect measure(s) (e.g. risk ratio, mean difference) used in the synthesis or presentation of results.	6
Synthesis methods	13a	Describe the processes used to decide which studies were eligible for each synthesis (e.g. tabulating the study intervention characteristics and comparing against the planned groups for each synthesis (item #5)).	6,7
	13b	Describe any methods required to prepare the data for presentation or synthesis, such as handling of missing summary statistics, or data conversions.	6,7
	13c	Describe any methods used to tabulate or visually display results of individual studies and syntheses.	6,7
	13d	Describe any methods used to synthesize results and provide a rationale for the choice(s). If meta-analysis was performed, describe the model(s), method(s) to identify the presence and extent of statistical heterogeneity, and software package(s) used.	6,7

Section and Topic	Item #	Checklist item	Location where item is reported
	13e	Describe any methods used to explore possible causes of heterogeneity among study results (e.g. subgroup analysis, meta-regression).	7
	13f	Describe any sensitivity analyses conducted to assess robustness of the synthesized results.	7
Reporting bias assessment	14	Describe any methods used to assess risk of bias due to missing results in a synthesis (arising from reporting biases).	7
Certainty assessment	15	Describe any methods used to assess certainty (or confidence) in the body of evidence for an outcome.	7
**RESULTS**			
Study selection	16a	Describe the results of the search and selection process, from the number of records identified in the search to the number of studies included in the review, ideally using a flow diagram.	7
	16b	Cite studies that might appear to meet the inclusion criteria, but which were excluded, and explain why they were excluded.	7
Study characteristics	17	Cite each included study and present its characteristics.	7-9
Risk of bias in studies	18	Present assessments of risk of bias for each included study.	12
Results of individual studies	19	For all outcomes, present, for each study: (a) summary statistics for each group (where appropriate) and (b) an effect estimate and its precision (e.g. confidence/credible interval), ideally using structured tables or plots.	9-11
Results of syntheses	20a	For each synthesis, briefly summarise the characteristics and risk of bias among contributing studies.	9-11
	20b	Present results of all statistical syntheses conducted. If meta-analysis was done, present for each the summary estimate and its precision (e.g. confidence/credible interval) and measures of statistical heterogeneity. If comparing groups, describe the direction of the effect.	9-11
	20c	Present results of all investigations of possible causes of heterogeneity among study results.	9-11
	20d	Present results of all sensitivity analyses conducted to assess the robustness of the synthesized results.	9-11
Reporting biases	21	Present assessments of risk of bias due to missing results (arising from reporting biases) for each synthesis assessed.	12
Certainty of evidence	22	Present assessments of certainty (or confidence) in the body of evidence for each outcome assessed.	12
**DISCUSSION**			
Discussion	23a	Provide a general interpretation of the results in the context of other evidence.	12, 13
	23b	Discuss any limitations of the evidence included in the review.	14,15
	23c	Discuss any limitations of the review processes used.	15
	23d	Discuss implications of the results for practice, policy, and future research.	13, 14
**OTHER INFORMATION**			
Registration and protocol	24a	Provide registration information for the review, including register name and registration number, or state that the review was not registered.	3
	24b	Indicate where the review protocol can be accessed, or state that a protocol was not prepared.	3
	24c	Describe and explain any amendments to information provided at registration or in the protocol.	3
Support	25	Describe sources of financial or non-financial support for the review,	15

Section and Topic	Item #	Checklist item	Location where item is reported
		and the role of the funders or sponsors in the review.	
Competing interests	26	Declare any competing interests of review authors.	15
Availability of data, code and other materials	27	Report which of the following are publicly available and where they can be found: template data collection forms; data extracted from included studies; data used for all analyses; analytic code; any other materials used in the review.	15

Table S2. Search strategy

Base	Search strategy
PubMed	(<br>"S100 Calcium Binding Protein beta Subunit"[Mesh]<br>OR "S100B protein"[tiab]<br>OR "S100B"[tiab]<br>OR "S 100B"[tiab]<br>OR "S100 B"[tiab]<br>OR "S-100B"[tiab]<br>OR "S-100 B"[tiab]<br>OR "S100 beta"[tiab]<br>OR "S-100 beta"[tiab]<br>OR "S100β"[tiab]<br>OR "S-100β"[tiab]<br>OR "S-100 protein"[tiab]<br>OR "S100 protein"[tiab]<br>OR "S100 calcium binding protein B"[tiab]<br>OR "S100 calcium-binding protein B"[tiab]<br>)<br>AND<br>(<br>"Heart Arrest"[Mesh]<br>OR "Heart Arrest, Induced"[Mesh]<br>OR "Cardiopulmonary Resuscitation"[Mesh]<br>OR "Out-of-Hospital Cardiac Arrest"[tiab]<br>OR "out-of-hospital cardiac arrest"[tiab]<br>OR "out of hospital cardiac arrest"[tiab]<br>OR OHCA[tiab]<br>OR "In-Hospital Cardiac Arrest"[tiab]<br>OR "in-hospital cardiac arrest"[tiab]<br>OR "in hospital cardiac arrest"[tiab]<br>OR IHCA[tiab]<br>OR "cardiac arrest"[tiab]<br>OR "heart arrest"[tiab]<br>OR "post-cardiac arrest"[tiab]<br>OR "post cardiac arrest"[tiab]

	OR "postresuscitation"[tiab]<br>OR "post-resuscitation"[tiab]<br>OR "cardiopulmonary resuscitation"[tiab]<br>OR CPR[tiab]<br>OR "return of spontaneous circulation"[tiab]<br>OR ROSC[tiab]<br>OR "sudden cardiac death"[tiab]<br>)<br>AND<br>(<br>"Adult"[Mesh]<br>OR "Young Adult"[Mesh]<br>OR "Middle Aged"[Mesh]<br>OR "Aged"[Mesh]<br>OR "Aged, 80 and over"[Mesh]<br>OR adult[tiab]<br>OR adults[tiab]<br>OR "adult patients"[tiab]<br>OR "adult population"[tiab]<br>)<br>NOT<br>(<br>animals[mh]<br>NOT humans[mh]<br>)
EMBASE	1. 's100 calcium binding protein b'/exp<br>2. 's100b protein'/exp<br>3. (S100B OR 'S 100B' OR 'S100 B' OR 'S-100B' OR 'S-100 B'<br>OR 'S100 beta' OR 'S-100 beta' OR 'S100β' OR 'S-100β'<br>OR 's100 calcium binding protein b' OR 's100 calcium-binding protein b'<br>):ti,ab,kw<br>4. 1 OR 2 OR 3<br><br>5. 'heart arrest'/exp<br>6. 'cardiopulmonary resuscitation'/exp<br>7. 'sudden cardiac death'/exp<br>8. ('out of hospital cardiac arrest' OR 'out-of-hospital cardiac arrest' OR OHCA<br>OR 'in hospital cardiac arrest' OR 'in-hospital cardiac arrest' OR IHCA<br>OR 'cardiac arrest' OR 'heart arrest'<br>OR 'post cardiac arrest' OR 'post-cardiac arrest'<br>OR postresuscitation OR 'post-resuscitation'<br>OR CPR OR 'cardiopulmonary resuscitation'<br>OR 'return of spontaneous circulation' OR ROSC<br>):ti,ab,kw<br>9. 5 OR 6 OR 7 OR 8

	10. 'adult'/exp OR 'young adult'/exp OR 'middle aged'/exp OR 'aged'/exp OR 'very elderly'/exp<br>11. (adult OR adults OR 'adult patients' OR 'adult population'):ti,ab,kw<br>12. 10 OR 11<br><br>13. 4 AND 9 AND 12<br><br>14. [humans]/lim<br>15. 13 AND 14<br><br>16. [english]/lim<br>17. 15 AND 16
SCOPUS	(<br>TITLE-ABS-KEY(<br>S100B OR "S 100B" OR "S100 B" OR "S-100B" OR "S-100 B"<br>OR "S100 beta" OR "S-100 beta" OR S100β OR "S-100β"<br>OR "S100 protein" OR "S-100 protein"<br>OR "S100 calcium binding protein B"<br>OR "S100 calcium-binding protein B"<br>)<br>)<br>AND<br>(<br>TITLE-ABS-KEY(<br>"cardiac arrest" OR "heart arrest"<br>OR "out-of-hospital cardiac arrest" OR "out of hospital cardiac arrest" OR OHCA<br>OR "in-hospital cardiac arrest" OR "in hospital cardiac arrest" OR IHCA<br>OR "post cardiac arrest" OR "post-cardiac arrest"<br>OR postresuscitation OR "post-resuscitation"<br>OR "cardiopulmonary resuscitation" OR CPR<br>OR "return of spontaneous circulation" OR ROSC<br>OR "sudden cardiac death"<br>)<br>)<br>AND<br>(<br>TITLE-ABS-KEY(<br>adult OR adults OR "adult population" OR "adult patients"<br>)<br>)<br>AND (LIMIT-TO (LANGUAGE , "English"))<br>AND (LIMIT-TO (DOCTYPE , "ar"))
Web of Science	TS=(<br>(

	S100B OR "S 100B" OR "S100 B" OR "S-100B" OR "S-100 B"<br>OR "S100 beta" OR "S-100 beta" OR S100β OR "S-100β"<br>OR "S100 protein" OR "S-100 protein"<br>OR "S100 calcium binding protein B"<br>OR "S100 calcium-binding protein B"<br>)<br>AND<br>(<br>"cardiac arrest" OR "heart arrest"<br>OR "out-of-hospital cardiac arrest" OR "out of hospital cardiac arrest" OR OHCA<br>OR "in-hospital cardiac arrest" OR "in hospital cardiac arrest" OR IHCA<br>OR "post cardiac arrest" OR "post-cardiac arrest"<br>OR postresuscitation OR "post-resuscitation"<br>OR "cardiopulmonary resuscitation" OR CPR<br>OR "return of spontaneous circulation" OR ROSC<br>OR "sudden cardiac death"<br>)<br>AND<br>(<br>adult OR adults OR "adult patients" OR "adult population"<br>)<br>)<br>Refined by:<br>DOCUMENT TYPES: (ARTICLE)<br>LANGUAGES: (ENGLISH)<br>Timespan: 1900–2025<br>Indexes: SCI-EXPANDED, SSCI, ESCI
COCHRANE CENTRAL (CENTRAL)	(<br>(S100B OR "S 100B" OR "S100 B" OR "S-100B" OR "S-100 B"<br>OR "S100 beta" OR "S-100 beta" OR S100β OR "S-100β"<br>OR "S100 protein" OR "S-100 protein"<br>OR "S100 calcium binding protein B"<br>OR "S100 calcium-binding protein B")<br>:ti,ab,kw<br>)<br>AND<br>(<br>("cardiac arrest" OR "heart arrest"<br>OR "out-of-hospital cardiac arrest" OR "out of hospital cardiac arrest" OR OHCA<br>OR "in-hospital cardiac arrest" OR "in hospital cardiac arrest" OR IHCA<br>OR "post cardiac arrest" OR "post-cardiac arrest"<br>OR postresuscitation OR "post-resuscitation"<br>OR "cardiopulmonary resuscitation" OR CPR

	OR "return of spontaneous circulation" OR ROSC<br>OR "sudden cardiac death")<br>:ti,ab,kw<br>)
CINAHL	S1: (MH "S100 Proteins+")<br>OR (S100B OR "S 100B" OR "S100 B" OR "S-100B" OR "S-100 B"<br>OR "S100 beta" OR "S-100 beta" OR S100β OR "S-100β"<br>OR "S100 protein" OR "S-100 protein"<br>OR "S100 calcium binding protein B"<br>OR "S100 calcium-binding protein B")<br>S2: (MH "Heart Arrest+")<br>OR (MH "Cardiopulmonary Resuscitation+")<br>OR ("cardiac arrest" OR "heart arrest"<br>OR "out-of-hospital cardiac arrest" OR "out of hospital cardiac arrest" OR OHCA<br>OR "in-hospital cardiac arrest" OR "in hospital cardiac arrest" OR IHCA<br>OR "post cardiac arrest" OR "post-cardiac arrest"<br>OR postresuscitation OR "post-resuscitation"<br>OR "cardiopulmonary resuscitation" OR CPR<br>OR "return of spontaneous circulation" OR ROSC<br>OR "sudden cardiac death")<br>S3: S1 AND S2<br>Limiters:<br>English Language<br>Humans<br>Age Groups: Adult (19–44), Middle Aged (45–64), Aged (65+)
Google Scholar	("S100 protein" OR "S-100 protein" OR "S100 calcium binding protein B" OR "S100 calcium-binding protein B")<br>AND<br>("cardiac arrest" OR OHCA OR IHCA OR CPR OR ROSC OR "post-cardiac arrest")

Table S3. Baseline characteristics of included trials.

Study	Country	Study design	Study group	Population	Age, years	Sex, male	Shockable primary rhythm (%)	Witnessed arrest (%)	Bystander CPR (%)	OHCA (%)	NOS score
Akin et al., 2021	Germany	Prospective observational registry	SHD	83	68 (12)	34	54	65	52	68 (100)	9
			Non-SHD	168	59 (13)	134	132	149	120	168 (100)	
			GNO	102	57 (13)	81	81	92	77	102 (100)	
			PNO	145	66 (13)	116	105	122	95	145 (100)	
Choi et al., 2016	Korea	Prospective observational study	GNO	46	50.57 (16.0)	37 (80.4)	33 (71.7)	38 (82.6)	25 (54.3)	46 (100)	7
			PNO	73	55.84 (15.2)	44 (60.3)	15 (20.5)	53 (72.6)	30 (41.1)	73 (100)	
Choi et al., 2021	Korea	Retrospective cohort study	GNO	41	56 (41-62)	32 (78.1)	28 (68.3)	31 (75.6)	29 (70.7)	41 (100)	8
			PNO	117	61 (51-73)	71 (60.7)	13 (11.1)	78 (66.7)	85 (72.7)	117 (100)	
Deye et al., 2020	France	Prospective single-center study	GNO	109	55 (45-67)	84 (77.1)	80 (73.4)	103 (94.5)	87 (79.8)	85 (78.0)	8
			PNO	221	63 (52-76)	146 (66.1)	45 (20.4)	209 (94.6)	139 (62.9)	166 (75.1)	
Duez et al., 2018	Denmark	Retrospective analysis of prospectively collected data	GNO	79	NS	NS	NS	NS	NS	79 (100)	8
			PNO	36	NS	NS	NS	NS	NS	36 (100)	
Einav et al., 2012	Izrael		GNO	26	NS	NS	NS	NS	NS	26 (100)	8

		Prospective single-center study	PNO	169	NS	NS	NS	NS	NS	169 (100)	
Einav et al., 2013	Izrael	Prospective observational study	SHD	32	65.8 (14.2)	26 (81.3)	19 (59.4)	NS	NS	32 (100)	8
			Non-SHD	126	76.4 (15.2)	78 (61.9)	15 (11.9)	NS	NS	126 (100)	
Elmer et al., 2016	USA	Prospective observational study	SHD	39	56 (43-56)	21 (53.8)	24 (63.2)	NS	NS	28 (71.8)	8
			Non-SHD	47	56 (45-56)	29 (61.7)	15 (31.9)	NS	NS	31 (66.0)	
Grubb et al., 2007	UK	Prospective observational study	SHD	56	NS	NS	NS	NS	NS	56 (100)	9
			Non-SHD	77	NS	NS	NS	NS	NS	77 (100)	
Huang et al., 2016	Taiwan	Prospective observational study	SHD	45	68.79 (15.24)	29 (64.4)	9 (20.0)	36 (80.0)	NS	45 (100)	8
			Non-SHD	54	72.61 (12.41)	29 (53.7)	5 (9.3)	37 (68.5)	NS	54 (100)	
Huesgen et al., 2021	USA	Prospective observational study	GNO	10	55.56 (14.37)	7 (70.0)	8 (80.0)	9 (90.0)	NS	10 (100)	8
			PNO	12	64.58 (14.10)	8 (66.7)	2 (16.7)	10 (83.3)	NS	12 (100)	
Jang et al., 2019	Korea	Prospective observational study	GNO	30	48.5 (41.5 – 54.5)	25 (83.3)	22 (73.3)	28 (93.3)	13 (43.3)	30 (100)	8
			PNO	67	52 (41-64)	39 (58.2)	14 (20.9)	51 (76.1)	16 (23.9)	67 (100)	
Jenei et al., 2013	Hungary	Prospective observational study	SHD	24	57 (47-66)	20	NS	NS	NS	17	8
			Non-SHD	22	66 (60-69)	18	NS	NS	NS	19	
Jeon et al., 2025	Korea	Retrospective study	GNO	46	55 (37.8-66.5)	37 (80.4)	27 (58.7)	37 (80.4)	38 (82.6)	46 (100)	9
			PNO	65	57 (43.5-70)	44 (67.7)	6 (9.2)	27 (41.5)	40 (61.5)	65 (100)	

Kim et al., 2014	Korea	Prospective observational study	GNO	18	48.5 (42.5- 58)	16 (88.9)	11 (61.1)	16 (88.9)	7 (38.9)	18 (100)	7
			PNO	22	58.0 (47.8 – 67.3)	15 (68.2)	6 (27.3)	15 (68.2)	8 (36.4)	22 (100)	
Kim et al., 2017	Korea	Retrospective observational study	GNO	12	60.5 (22-79)	6 (50.0)	3 (25.0)	12 (100)	5 (41.7)	12 (100)	7
			PNO	17	71 (45-86)	13 (76.5)	2 (11.8)	13 (76.5)	4 (23.5)	17 (100)	
Kim et al., 2023	Korea	Prospective observational study	GNO	13	NS	NS	NS	NS	NS	13 (100)	8
			PNO	43	NS	NS	NS	NS	NS	43 (100)	
Kleissner et al., 2021	Czechia	Prospective, single-center study	GNO	30	59 (13)	23 (76.7)	28 (93.3)	25 (83.3)	25 (83.3)	30 (100)	8
			PNO	18	64 (11)	17 (94.4)	10 (55.6)	18 (100)	15 (83.3)	18 (100)	
Larsson et al., 2014	Sweden	Prospective observational study	GNO	57	63 (14)	38 (66.7)	35 (52.2)	52 (91.2)	34 (59.6)	38 (66.7)	9
			PNO	68	69 (12)	45 (66.2)	22 (32.3)	56 (82.3)	39 (57.3)	45 (66.2)	
Mörtberg et al., 2011	Sweden	Prospective observational study	GNO	15	60 (22-78)	12 (80.0)	10 (66.7)	13 (86.7)	9 (60.0)	13 (86.7)	7
			PNO	16	68.5 (25-84)	9 (56.3)	6 (37.5)	12 (75.0)	9 (56.3)	14 (87.5)	
Oda et al., 2012	Japan	Prospective observational study	GNO	7	50 (41-71)	5 (71.4)	6 (85.7)	6 (85.7)	4 (57.1)	7 (100)	8
			PNO	18	67 (51-69)	14 (77.8)	9 (50.0)	4 (22.2)	9 (50.0)	18 (100)	
Oh et al., 2021	Korea	Prospective observational study	GNO	20	48 (34-65.6)	12 (60.0)	13 (65.0)	16 (80.0)	15 (75.0)	15 (75.0)	8
			PNO	34	60 (44.8-72)	25 (73.5)	4 (11.8)	21 (61.8)	18 (52.9)	32 (94.1)	
Ok et al., 2016	Türkiye		GNO	19	53.6 (8.4)	17 (89.5)	6 (31.6)	NS	NS	0 (0)	7

		Prospective observational study	PNO	11	68.6 (9.5)	8 (72.7)	3 (27.3)	NS	NS	0 (0)	
Park et al., 2019	Korea	Retrospective study	GNO	39	53 (40-59)	26 (66.7)	23 (59.0)	19 (48.7)	24 (61.5)	39 (100)	8
			PNO	63	57 (48-68)	46 (73.0)	12 (19.0)	28 (44.4)	33 (52.4)	63 (100)	
Petermichl et al., 2021	Germany	Retrospective study	GNO	17	NS	NS	NS	NS	NS	17 (100)	8
			PNO	46	NS	NS	NS	NS	NS	46 (100)	
			SHD	21	NS	NS	NS	NS	NS	21 (100)	
			Non-SHD	42	NS	NS	NS	NS	NS	42 (100)	
Pfeifer et al., 2005	Germany	Prospective cohort study	GNO	27	63.2 (29-87)	NS	18 (66.7)	21 (77.8)	20 (74.1)	NS	9
			PNO	70	63 (17-90)	NS	28 (40.0)	38 (54.3)	22 (31.4)	NS	
Rana et al., 2012	Germany	Prospective cohort study	GNO	51	60.9 (12.5)	40	NS	NS	NS	51 (100)	8
			PNO	46	70.6 (13.2)	32	NS	NS	NS	46 (100)	
Rundgren et al., 2009	Sweden	Prospective cohort study	GNO	57	NS	NS	NS	NS	NS	NS	8
			PNO	50	NS	NS	NS	NS	NS	NS	
Ryczek et al., 2022	Poland	Prospective observational study	GNO	22	60 (45-70)	15 (68.2)	20 (90.9)	NS	20 (90.9)	22 (100)	8
			PNO	60	67 (62-76.5)	41 (68.3)	34 (56.7)	NS	31 (51.7)	60 (100)	
Shinozaki et al., 2009	Japan	Prospective observational study	GNO	13	59 (12)	13 (100)	NS	13 (100)	NS	11 (84.6)	8
			PNO	67	67 (15)	35 (52.2)	NS	48 (71.6)	NS	47 (70.1)	
Song et al., 2010	Korea		SHD	46	63 (14.9)	26 (56.5)	7 (15.2)	29 (63.0)	9 (19.6)	46 (100)	7

		Prospective observational study	Non-SHD	105	59 (17.5)	71 (67.6)	10 (9.5)	58 (55.2)	4 (3.8)	105 (100)	
Song et al., 2023	Korea	Prospective observational study	GNO	46	53.3 (15.5)	39 (84.8)	NS	32 (69.6)	38 (82.6)	46 (100)	8
			PNO	54	58.3 (16.2)	39 (72.2)	NS	32 (59.3)	34 (63.0)	53 (100)	
Stammet et al., 2013	Luxembourg	Prospective observational study	GNO	41	61 (29-82)	34 (82.9)	36 (87.8)	NS	NS	41 (100)	8
			PNO	34	69 (38-83)	23 (67.6)	13 (38.2)	NS	NS	34 (100)	
Stammet et al., 2017	Luxembourg	Retrospective analysis of prospectively collected data	GNO	389	NS	NS	NS	NS	NS	389 (100)	8
			PNO	295	NS	NS	NS	NS	NS	295 (100)	
Strate et al., 2025	The Netherlands	Retrospective analysis of prospectively collected data	GNO	22	NS	NS	NS	NS	NS	22 (100)	8
			PNO	18	NS	NS	NS	NS	NS	18 (100)	
Tat et al., 2019	Romania	Prospective observational study	SHD	12	60.5 (45-66.7)	9 (75.0)	10 (83.3)	NS	NS	12 (100)	7
			Non-SHD	28	69 (66-76.7)	19 (67.9)	2 (7.1)	NS	NS	28 (100)	
Wennervirt et al., 2009	Finland	Prospective observational study	GNO	21	55 (24-74)	16 (76.2)	NS	NS	NS	21 (100)	8
			PNO	9	60 (26-77)	8 (88.9)	NS	NS	NS	9 (100)	
Wiberg et al., 2017	Denmark	Prospective observational study	SHD	19	33 (16-50)	12 (63.2)	NS	12 (63.2)	2 (10.5)	19 (100)	8
			Non-SHD	15	39 (21-71)	12 (92.3)	NS	4 (26.7)	2 (13.3)	15 (100)	
Wurm et al., 2022	Austria	Post hoc analysis of a prospectively collected	GNO	21	56 (43-66)	16 (76.2)	19 (90.5)	19 (90.5)	NS	21 (100)	8
			PNO	49	60 (49-70)	37 (75.5)	35 (71.4)	37 (75.5)	NS	49 (100)	

		single-center study									
Zellner et al., 2013	Germany	Retrospective study	GNO	61	61 (14)	52 (83.6)	50 (80.2)	52 (85.2)	37 (60.7)	57 (93.4)	7
			PNO	62	63 (15)	46 (74.2)	31 (50.8)	40 (64.5)	31 (50.8)	53 (85.5)	

Legend: CPR = Cardiopulmonary resuscitation; GNO = Good neurological outcome; NOS = Newcastle Ottawa Scale; NS = not specified; OHCA = Out-of-hospital cardiac arrest; PNO = Poor neurological outcome; SHD = Survival to hospital discharge.

Table S4. Summary of S100B concentrations and standardized mean differences between patients with good and poor 6-month neurological outcomes at baseline and 24, 48, 72, and 96 hours after cardiac arrest.

Measurement Period after Cardiac Arrest	No. of Studies	Mean (SD)		Events		Heterogeneity between Trials		p-Value for Differences across Groups
		Good outcome	Poor outcome	SMD	95% CI	p-Value	I^2 statistics	
**S100B on day 0**								
All Trials	15	0.717 (0.557)	1.805 (1.547)	-1.78	-2.25 to -1.31	<0.001	89%	<0.001
OHCA	10	0.516 (0.484)	1.732 (1.848)	-1.09	-1.49 to -0.70	<0.001	98%	<0.001
Asian region	7	0.482 (0.601)	1.933 (1.882)	-1.43	-1.97 to -0.90	<0.001	94%	<0.001
Europe/USA	8	0.830 (0.498)	1.685 (1.135)	-0.91	-1.29 to -0.54	<0.001	98%	<0.001
**S100B on 24h**								
All Trials	15	0.146 (0.225)	0.734 (1.010)	-2.41	-3.01 to -1.81	<0.001	94%	<0.001
OHCA	10	0.517 (0.484)	1.732 (1.848)	-1.09	-1.49 to -0.70	<0.001	98%	<0.001
Asian region	7	0.482 (0.601)	1.939 (1.882)	-1.43	-1.97 to -0.90	<0.001	94%	<0.001
Europe/USA	8	0.830 (0.498)	1.685 (1.135)	-0.91	-1.29 to -0.54	<0.001	98%	<0.001
**S100B on 48h**								
All Trials	12	0.109 (0.113)	0.341 (0.495)	-3.07	-3.80 to -2.34	<0.001	95%	<0.001
OHCA	9	0.112 (0.133)	0.616 (1.137)	-0.48	-0.57 to -0.38	<0.001	99%	<0.001
Asian region	5	0.217 (0.189)	1.0.17 (1.177)	-0.91	-1.22 to -0.60	<0.001	95%	<0.001
Europe/USA	7	0.129 (0.141)	0.468 (0.957)	-0.37	-0.45 to -0.28	<0.001	99%	<0.001
**S100B on 72h**								
All Trials	10	0.109 (0.113)	0.341 (0.495)	-2.92	-3.78 to -2.07	<0.001	96%	<0.001
OHCA	7	0.100 (0.115)	0.321 (0.541)	-3.12	-4.32 to -1.93	<0.001	97%	<0.001
Asian region	3	0.104 (0.051)	0.437 (0.214)	-2.16	-2.48 to -1.85	0.88	0%	<0.001
Europe/USA	7	0.111 (0.122)	0.317 (0.541)	-3.28	-4.44 to -2.12	<0.001	96%	<0.001
**S100B on 96h**								
All Trials	3	0.175 (0.125)	1.019 (0.631)	-2.35	-2.62 to -2.08	0.37	0%	<0.001
OHCA	2	0.261 (0.111)	1.252 (0.511)	-2.22	-2.55 to -1.90	0.75	0%	<0.001
Asian region	1	0.149 (0.047)	0.773 (0.348)	-2.35	-3.17 to -1.52	NA	NA	<0.001
Europe/USA	2	0.179 (0.134)	1.037 (0.645)	-2.38	-2.80 to -1.97	0.16	49%	<0.001

Table S5. Summary of S100B concentrations and standardized mean differences between patients with good and poor hospital discharge neurological outcomes at baseline and 24, 48, 72, and 96 hours after cardiac arrest.

Measurement Period after Cardiac Arrest	No. of Studies	Mean (SD)		Events		Heterogeneity between Trials		p-Value for Differences across Groups
		Good outcome	Poor outcome	SMD	95% CI	p-Value	I^2 statistics	
**S100B on day 0**								
All Trials	9	1.349 (1.105)	4.506 (3.683)	-1.65	-2.54 to -0.76	<0.001	95%	<0.001
OHCA	8	1.426 (1.107)	4.529 (3.685)	-1.71	-2.68 to -0.73	<0.001	96%	<0.001
Asian region	4	1.168 (1.129)	3.610 (3.482)	-1.79	-3.42 to -0.17	<0.001	97%	0.03
Europe/USA	5	1.555 (1.044)	5.688 (3.612)	-1.54	-2.68 to -0.39	<0.001	94%	0.008
**S100B on 24h**								
All Trials	7	0.294 (0.316)	2.714 (3.397)	-1.94	-2.72 to -1.17	<0.001	93%	<0.001
OHCA	5	0.197 (0.154)	2.865 (3.644)	-2.05	-2.96 to -1.14	<0.001	94%	<0.001
Asian region	3	0.172 (0.141)	3.015 (4.849)	-1.88	-3.31 to -0.45	<0.001	97%	0.01
Europe/USA	4	0.467 (0.403)	2.456 (1.095)	-2.01	-2.90 to -1.12	<0.001	88%	<0.001
**S100B on 48h**								
All Trials	5	0.176 (0.113)	1.737 (1.587)	-2.00	-3.09 to -0.91	<0.001	95%	<0.001
OHCA	4	0.139 (0.082)	1.521 (1.770)	-1.41	-2.08 to -0.75	<0.001	85%	<0.001
Asian region	2	0.162 (0.094)	1.502 (1.857)	-1.08	-1.97 to -0.20	0.003	89%	0.02
Europe/USA	3	0.229 (0.178)	1.976 (1.217)	-2.65	-4.41 to -0.90	<0.001	95%	0.003
**S100B on 72h**								
All Trials	5	0.154 (0.108)	1.466 (1.229)	-2.76	-3.71 to -1.82	<0.001	94%	<0.001
OHCA	4	0.125 (0.078)	1.262 (1.191)	-2.21	-2.91 to -1.51	<0.001	89%	<0.001
Asian region	1	0.193 (0.068)	1.703 (1.308)	-1.45	-1.90 to -1.01	NA	NA	<0.001
Europe/USA	4	0.146 (0.114)	1.432 (1.216)	-3.10	-4.09 to -2.11	<0.001	92%	<0.001
**S100B on 96h**								
All Trials	2	0.177 (0.136)	1.886 (1.321)	-3.54	-6.91 to -0.18	<0.001	97%	0.04
OHCA	1	0.013 (0.012)	0.367 (0.221)	-1.84	-2.49 to -1.19	NA	NA	<0.001
Asian region	-	-	-	-	-	-	-	-
Europe/USA	2	0.177 (0.136)	1.886 (1.321)	-3.54	-6.91 to -0.18	<0.001	97%	0.04

Table S6. Summary of S100B concentrations and standardized mean differences between patients with good and poor 3-month neurological outcomes at baseline and 24, 48, 72, and 96 hours after cardiac arrest.

Measurement Period after Cardiac Arrest	No. of Studies	Mean (SD)		Events		Heterogeneity between Trials		p-Value for Differences across Groups
		Good outcome	Poor outcome	SMD	95% CI	p-Value	I^2 statistics	
**S100B on day 0**								
All Trials	2	0.411 (0.236)	1.707 (0.438)	-3.30	-4.08 to -2.51	0.02	80%	<0.001
OHCA	1	0.787 (0.252)	1.703 (0.35)	-2.86	-3.46 to -2.27	NA	NA	<0.001
Asian region	1	0.787 (0.252)	1.703 (0.35)	-2.86	-3.46 to -2.27	NA	NA	<0.001
Europe/USA	1	0.308 (0.062)	1.703 (0.462)	-3.66	-4.03 to -3.30	NA	NA	<0.001
**S100B on 24h**								
All Trials	2	0.119 (0.028)	0.691 (0.206)	-3.43	-3.95 to -2.90	0.15	51%	<0.001
OHCA	1	0.079 (0.012)	0.717 (0.20)	-3.79	-4.49 to -3.10	NA	NA	<0.001
Asian region	1	0.079 (0.012)	0.717 (0.20)	-3.79	-4.49 to -3.10	NA	NA	<0.001
Europe/USA	1	0.13 (0.02)	0.683 (0.208)	-3.23	-3.57 to -2.89	NA	NA	<0.001
**S100B on 48h**								
All Trials	2	0.101 (0.014)	0.487 (0.157)	-3.20	-3.54 to -2.87	0.28	13%	<0.001
OHCA	1	0.083 (0.01)	0.594 (0.173)	-3.51	-4.18 to -2.85	NA	NA	<0.001
Asian region	1	0.083 (0.01)	0.594 (0.173)	-3.51	-4.18 to -2.85	NA	NA	<0.001
Europe/USA	1	0.105 (0.01)	0.455 (0.137)	-3.11	-3.44 to -2.78	NA	NA	<0.001
**S100B on 72h**								
All Trials	1	0.076 (0.011)	0.324 (0.10)	-2.94	-3.55 to -2.34	NA	NA	<0.001
OHCA	1	0.076 (0.011)	0.324 (0.10)	-2.94	-3.55 to -2.34	NA	NA	<0.001
Asian region	1	0.076 (0.011)	0.324 (0.10)	-2.94	-3.55 to -2.34	NA	NA	<0.001
Europe/USA	-	-	-	-	-	-	-	-

Table S7. Summary of S100B concentrations and standardized mean differences between survivors and non-survivors to hospital discharge at 0, 24, 48, 72, and 96 hours after cardiac arrest.

Measurement Period after Cardiac Arrest	No. of Studies	Mean (SD)		Events		Heterogeneity between Trials		p-Value for Differences across Groups
		Survivors	Non-survivors	SMD	95% CI	p-Value	I^2 statistics	
**S100B on day 0**								
All Trials	8	1.077 (1.558)	1.967 (3.217)	-1.03	-1.83 to -0.22	<0.001	95%	0.01
OHCA	7	1.298 (1.627)	2.148 (3.317)	-0.93	-1.83 to -0.02	<0.001	95%	0.04
Asian region	2	0.934 (1.557)	3.160 (4.458)	-0.30	-0.63 to 0.03	0.76	0%	0.07
Europe/USA	6	1.124 (1.559)	1.273 (1.883)	-1.27	-2.31 to -0.22	<0.001	95%	0.02
**S100B on 24h**								
All Trials	6	0.101 (0.408)	0.814 (1.205)	-1.61	-1.89 to -1.34	0.63	0%	<0.001
OHCA	5	0.174 (0.433)	1.180 (1.708)	-1.72	-2.03 to -1.41	0.89	0%	<0.001
Asian region	-	-	-	-	-	-	-	-
Europe/USA	6	0.101 (0.408)	0.814 (1.205)	-1.61	-1.89 to -1.34	0.63	0%	<0.001
**S100B on 48h**								
All Trials	7	0.188 (0.283)	1.36 (1.840)	-1.58	-2.00 to -1.16	0.04	58%	<0.001
OHCA	6	0.251 (0.303)	1.806 (1.939)	-1.65	-2.22 to -1.08	0.02	66%	<0.001
Asian region	-	-	-	-	-	-	-	-
Europe/USA	7	0.135 (0.108)	0.659 (0.678)	-2.68	-3.43 to -1.92	<0.001	87%	<0.001
**S100B on 72h**								
All Trials	7	0.135 (0.108)	0.659 (0.678)	-2.68	-3.43 to -1.92	<0.001	87%	<0.001
OHCA	6	0.153 (0.102)	0.827 (0.665)	-2.71	-3.64 to -1.78	<0.001	89%	<0.001
Asian region	-	-	-	-	-	-	-	-
Europe/USA	7	0.135 (0.108)	0.659 (0.678)	-2.68	-3.43 to -1.92	<0.001	87%	<0.001
**S100B on 96h**								
All Trials	1	0.057 (0.126)	0.379 (0.22)	-1.64	-2.24 to -1.04	NA	NA	<0.001
OHCA	1	0.057 (0.126)	0.379 (0.22)	-1.64	-2.24 to -1.04	NA	NA	<0.001
Asian region	-	-	-	-	-	-	-	
Europe/USA	1	0.057 (0.126)	0.379 (0.22)	-1.64	-2.24 to -1.04	NA	NA	<0.001

Table S8. Diagnostic Performance of S100B at Different Time Points After Cardiac Arrest.

Metric	Value (95% CI)	Interpretation
**Baseline measurement**		
Summary Sensitivity	≈ 0.63 (0.53–0.72)	Moderate ability to detect poor neurological outcome
Summary Specificity	≈ 0.93 (0.86–0.97)	Very high specificity; false-positive results are rare
Pooled AUC (Summary ROC)	≈ 0.89 (0.85–0.92)	High overall diagnostic accuracy
**24 hours after cardiac arrest**		
Summary Sensitivity	0.50 (0.34–0.65)	Low–moderate ability to detect poor outcome
Summary Specificity	0.91 (0.83–0.96)	Very good specificity; few false-positive results
Pooled AUC (Summary ROC)	0.57 (0.49–0.65)	Poor diagnostic accuracy at this time point
**48 hours after cardiac arrest**		
Summary Sensitivity	0.48 (0.36–0.60)	Moderate ability to detect poor outcome
Summary Specificity	0.96 (0.91–0.99)	Very high specificity; minimal false-positive risk
Pooled AUC (Summary ROC)	0.81 (0.75–0.87)	Good overall diagnostic performance
**72 hours after cardiac arrest**		
Summary Sensitivity	0.35 (0.24–0.48)	Low ability to detect poor outcome
Summary Specificity	0.97 (0.91–0.99)	Very high specificity; almost no false positives
Pooled AUC (Summary ROC)	0.74 (0.67–0.81)	Moderate diagnostic accuracy

Table S9. S100B measured at different time points after ROSC (Outcome: Neurological outcome at 6 months).

Outcome	Time after CA	Studies	Study design	Risk of bias	Inconsistency	Indirectness	Imprecision	Publication bias	Certainty (GRADE)	Effect size (SMD, 95% CI)	Comments
Poor neurological outcome at 6 months	Baseline (0–6 h)	~13	Observational	Serious	Serious	Not serious	Not serious	Not detected	Moderate	≈ −2.0 (range −2.2 to −1.8)	Large effect size; substantial between-study heterogeneity
Poor neurological outcome at 6 months	24 h	~12	Observational	Serious	Serious	Not serious	Not serious	Not detected	Moderate	−2.41 (−3.01 to −1.81)	Large and precise effect; heterogeneity remains
Poor neurological outcome at 6 months	48 h	~10	Observational	Serious	Serious	Not serious	Not serious	Not detected	Moderate	−3.07 (−3.80 to −2.34)	Very large and consistent effect; most robust time point
Poor neurological outcome at 6 months	72 h	~9	Observational	Serious	Serious	Not serious	Not serious	Not detected	Moderate	−2.76 (−3.71 to −1.82)	Large effect persisting over time; heterogeneity persists
Poor neurological outcome at 6 months	96 h	3	Observational	Serious	Very serious	Not serious	Serious	Unclear	Very low	≈ −3.25 (wide CI)	Very limited data; wide CI; serious inconsistency

Study or Subgroup	CPC 1–2 Mean	CPC 1–2 SD	CPC 1–2 Total	CPC 3–5 Mean	CPC 3–5 SD	CPC 3–5 Total	Weight	Std. Mean Difference IV, Random, 95% CI
**1.2.1 Baseline**								
Choi 2016	1.18	1.8	46	3.3	4.3	73	11.6%	−0.59 [−0.97, −0.22]
Choi 2021	1.23	0.53	41	4.143	0.768	117	11.3%	−4.06 [−4.63, −3.48]
Einav 2012	2.305	0.965	26	8.528	2.002	169	11.4%	−3.26 [−3.79, −2.74]
Kleissner 2021	2.025	0.575	30	2.6	0.8	18	11.2%	−0.85 [−1.46, −0.24]
Ok 2016	0.45	0.56	19	3.05	3.4	11	10.8%	−1.22 [−2.03, −0.40]
Park 2019	1.048	0.273	39	2.37	0.82	63	11.4%	−1.97 [−2.45, −1.48]
Petermichl 2021	2.257	0.523	17	2.414	0.758	46	11.3%	−0.22 [−0.78, 0.34]
Ryczek 2022	0.439	0.083	22	1.61	0.639	60	11.2%	−2.11 [−2.70, −1.52]
Song 2010	1.71	0.91	3	3.87	4.71	148	9.9%	−0.46 [−1.60, 0.68]
**Subtotal (95% CI)**			**243**			**705**	**100.0%**	**−1.65 [−2.54, −0.76]**
Heterogeneity: $Tau^2 = 1.74$; $Chi^2 = 174.41$, df = 8 ($P < 0.00001$); $I^2 = 95\%$								
Test for overall effect: Z = 3.64 (P = 0.0003)								
**1.2.2 24h**								
Choi 2016	0.16	0.2	46	4.32	8.7	73	14.9%	−0.61 [−0.98, −0.23]
Choi 2021	0.118	0.028	41	3.08	1.153	117	14.6%	−2.96 [−3.45, −2.48]
Einav 2012	0.373	0.133	26	2.823	1.072	169	14.6%	−2.44 [−2.92, −1.96]
Ok 2016	0.33	0.59	19	0.84	1.51	11	13.5%	−0.49 [−1.24, 0.27]
Park 2019	0.243	0.093	39	1.38	0.685	63	14.5%	−2.08 [−2.57, −1.59]
Petermichl 2021	0.117	0.053	17	2.201	1.097	46	13.8%	−2.18 [−2.87, −1.50]
Pfeifer 2005	0.875	0.121	27	1.993	0.456	70	14.1%	−2.82 [−3.42, −2.22]
**Subtotal (95% CI)**			**215**			**549**	**100.0%**	**−1.94 [−2.72, −1.17]**
Heterogeneity: $Tau^2 = 1.02$; $Chi^2 = 92.01$, df = 6 ($P < 0.00001$); $I^2 = 93\%$								
Test for overall effect: Z = 4.90 (P < 0.00001)								
**1.2.3 48h**								
Choi 2016	0.13	0.1	46	1.24	2.2	73	20.8%	−0.64 [−1.02, −0.26]
Kleissner 2021	0.1	0.001	30	0.425	0.225	18	19.3%	−2.34 [−3.10, −1.58]
Park 2019	0.2	0.07	39	1.805	1.31	63	20.6%	−1.54 [−2.00, −1.09]
Petermichl 2021	0.093	0.021	17	2.005	1.665	46	20.0%	−1.32 [−1.93, −0.72]
Pfeifer 2005	0.458	0.058	27	2.356	0.508	70	19.3%	−4.34 [−5.10, −3.57]
**Subtotal (95% CI)**			**159**			**270**	**100.0%**	**−2.00 [−3.09, −0.91]**
Heterogeneity: $Tau^2 = 1.45$; $Chi^2 = 77.81$, df = 4 ($P < 0.00001$); $I^2 = 95\%$								
Test for overall effect: Z = 3.60 (P = 0.0003)								
**1.2.4 72h**								
Akin 2021	0.089	0.01	102	0.159	0.03	149	21.0%	−2.91 [−3.27, −2.55]
Einav 2012	0.23	0.07	26	2.233	0.852	169	20.5%	−2.51 [−2.99, −2.03]
Park 2019	0.193	0.068	39	1.703	1.308	63	20.6%	−1.45 [−1.90, −1.01]
Petermichl 2021	0.019	0.015	17	0.659	0.385	46	19.6%	−1.91 [−2.57, −1.26]
Pfeifer 2005	0.358	0.054	27	2.716	0.519	70	18.2%	−5.28 [−6.16, −4.40]
**Subtotal (95% CI)**			**211**			**497**	**100.0%**	**−2.76 [−3.71, −1.82]**
Heterogeneity: $Tau^2 = 1.08$; $Chi^2 = 67.40$, df = 4 ($P < 0.00001$); $I^2 = 94\%$								
Test for overall effect: Z = 5.72 (P < 0.00001)								
**1.2.5 96h**								
Petermichl 2021	0.013	0.012	17	0.367	0.221	46	50.4%	−1.84 [−2.49, −1.19]
Pfeifer 2005	0.28	0.043	27	2.884	0.574	70	49.6%	−5.28 [−6.15, −4.40]
**Subtotal (95% CI)**			**44**			**116**	**100.0%**	**−3.54 [−6.91, −0.18]**
Heterogeneity: $Tau^2 = 5.74$; $Chi^2 = 38.04$, df = 1 ($P < 0.00001$); $I^2 = 97\%$								
Test for overall effect: Z = 2.06 (P = 0.04)								

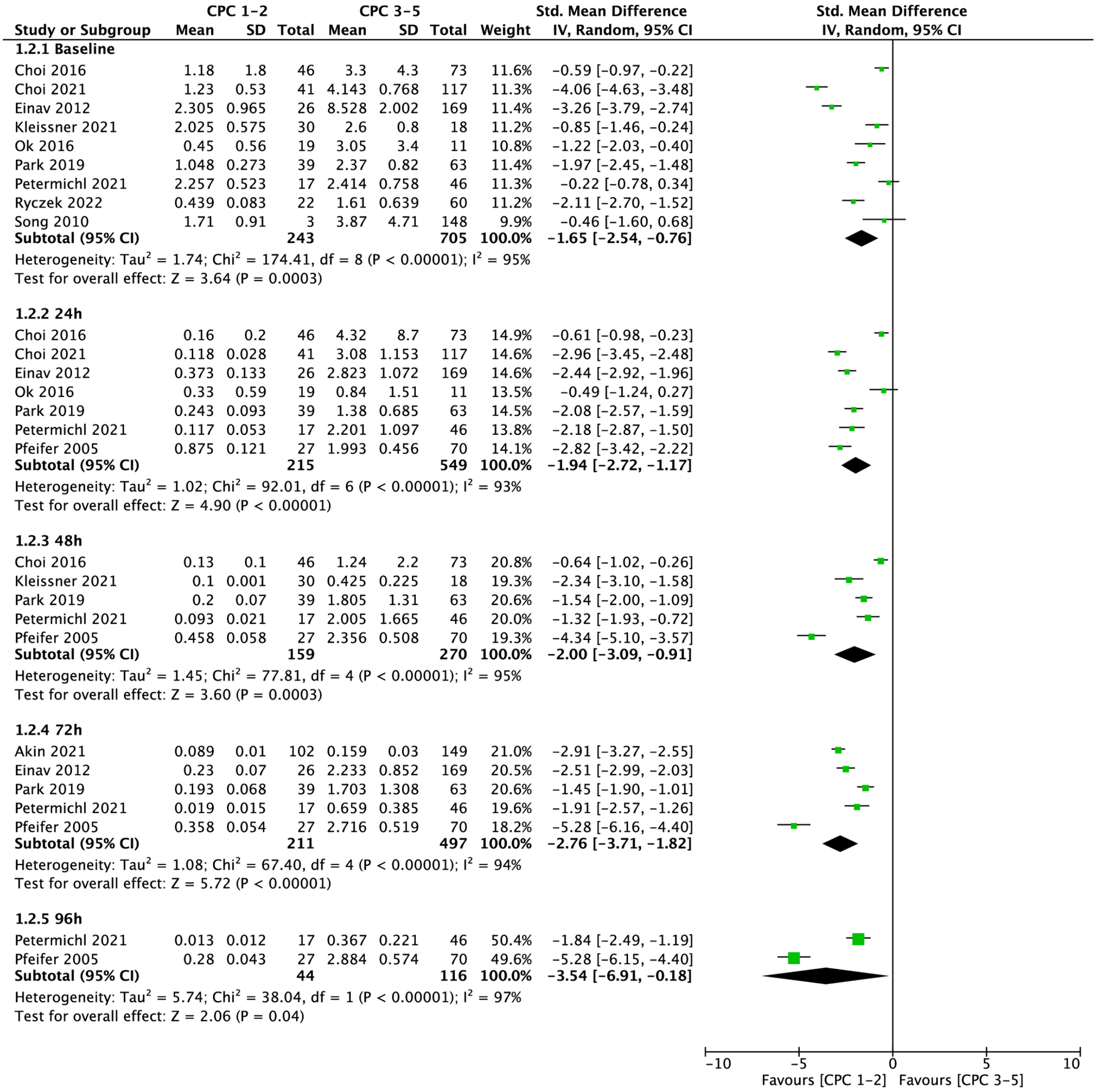

Figure S1. Forest plot presenting standardized mean differences (SMD) in S100B levels between patients with good and poor hospital discharge neurological outcomes at baseline and at 24, 48, 72, and 96 hours after cardiac arrest.

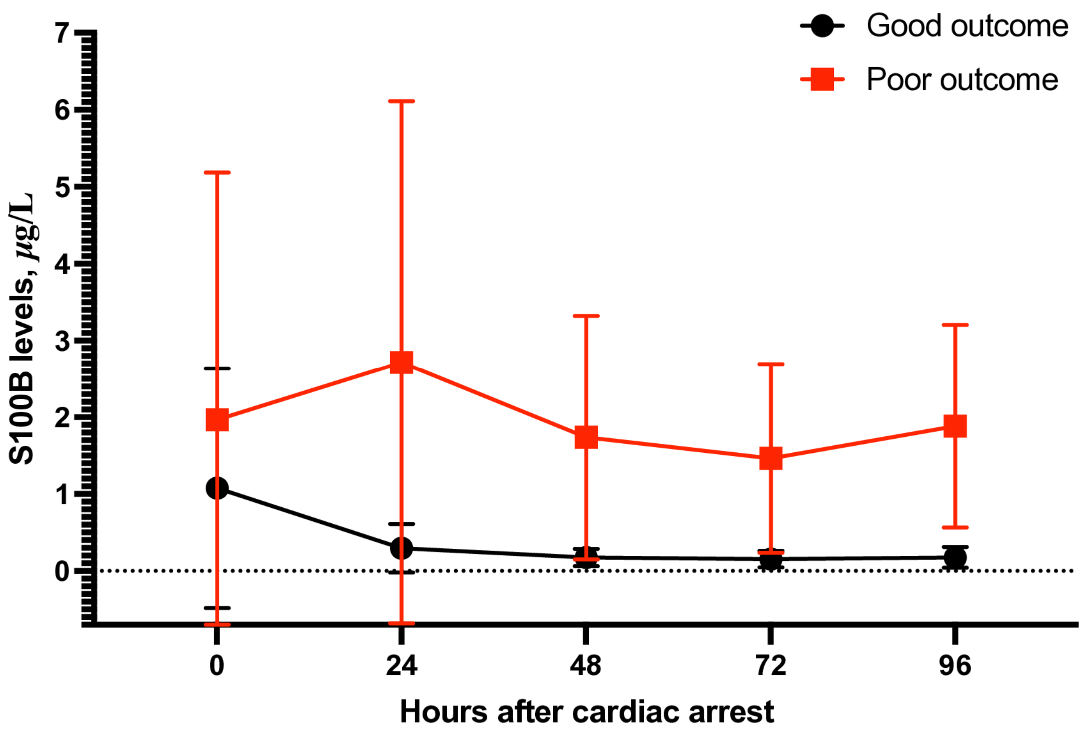

Figure S2. S100B levels (mean ± SD) at 0, 24, 48, 72, and 96 hours after cardiac arrest in patients with good and poor hospital discharge neurological outcomes.

Study or Subgroup	CPC 1–2 Mean	CPC 1–2 SD	CPC 1–2 Total	CPC 3–5 Mean	CPC 3–5 SD	CPC 3–5 Total	Weight	Std. Mean Difference IV, Random, 95% CI
**1.3.1 Baseline**								
Deye 2020	0.308	0.062	109	1.703	0.462	221	34.6%	−3.66 [−4.03, −3.30]
Jang 2019	0.787	0.252	30	1.719	0.35	67	33.0%	−2.86 [−3.46, −2.27]
Kang 2021	3.98	3.13	23	12.005	6.935	22	32.4%	−1.48 [−2.14, −0.81]
**Subtotal (95% CI)**			**162**			**310**	**100.0%**	**−2.69 [−3.93, −1.45]**
Heterogeneity: $Tau^2 = 1.13$; $Chi^2 = 32.66$, df = 2 (P < 0.00001); $I^2 = 94\%$								
Test for overall effect: Z = 4.24 (P < 0.0001)								
**1.3.2 24h**								
Deye 2020	0.13	0.02	109	0.683	0.208	221	58.7%	−3.23 [−3.57, −2.89]
Jang 2019	0.079	0.012	30	0.717	0.2	67	26.7%	−3.79 [−4.49, −3.10]
Kang 2021	2.818	1.938	23	22.933	7.068	22	14.6%	−3.85 [−4.87, −2.83]
**Subtotal (95% CI)**			**162**			**310**	**100.0%**	**−3.47 [−3.90, −3.05]**
Heterogeneity: $Tau^2 = 0.05$; $Chi^2 = 2.93$, df = 2 (P = 0.23); $I^2 = 32\%$								
Test for overall effect: Z = 16.05 (P < 0.00001)								
**1.3.3 48h**								
Deye 2020	0.105	0.01	109	0.455	0.137	221	47.9%	−3.11 [−3.44, −2.78]
Jang 2019	0.083	0.01	30	0.594	0.173	67	33.1%	−3.51 [−4.18, −2.85]
Kang 2021	1.478	0.838	23	23.078	6.923	22	19.1%	−4.35 [−5.46, −3.24]
**Subtotal (95% CI)**			**162**			**310**	**100.0%**	**−3.48 [−4.07, −2.88]**
Heterogeneity: $Tau^2 = 0.16$; $Chi^2 = 5.10$, df = 2 (P = 0.08); $I^2 = 61\%$								
Test for overall effect: Z = 11.46 (P < 0.00001)								
**1.3.4 72h**								
Jang 2019	0.076	0.011	30	0.324	0.1	67	56.2%	−2.94 [−3.55, −2.34]
Kang 2021	1.16	0.615	23	22.858	7.143	22	43.8%	−4.25 [−5.34, −3.16]
**Subtotal (95% CI)**			**53**			**89**	**100.0%**	**−3.52 [−4.79, −2.24]**
Heterogeneity: $Tau^2 = 0.66$; $Chi^2 = 4.25$, df = 1 (P = 0.04); $I^2 = 76\%$								
Test for overall effect: Z = 5.41 (P < 0.00001)								

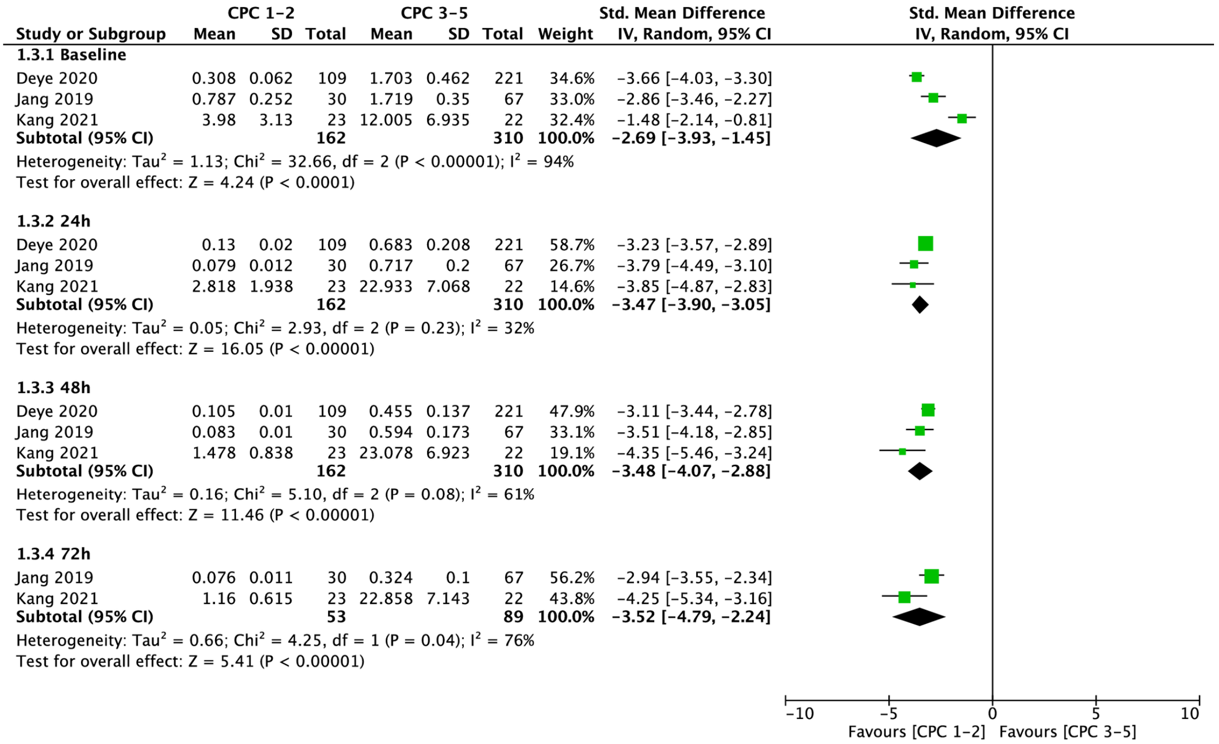

Figure S3. Forest plot presenting standardized mean differences in S100B levels between patients with good and poor 3-month neurological outcomes at baseline and at 24, 48, 72, and 96 hours after cardiac arrest.

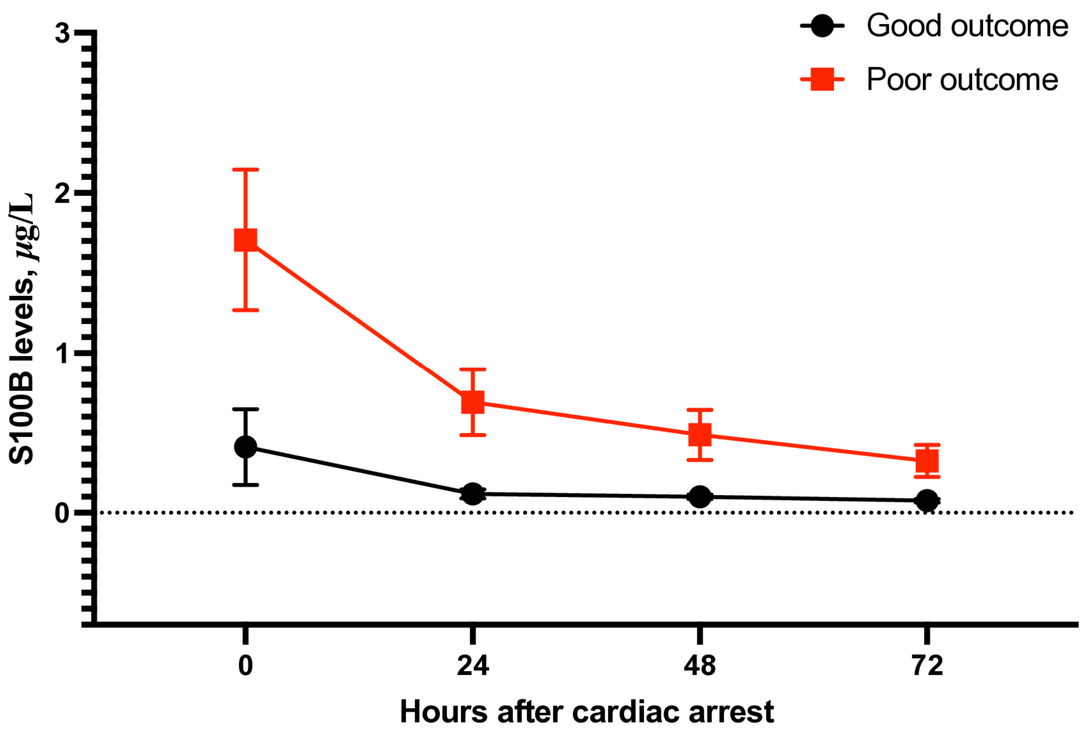

Figure S4. S100B levels (mean ± SD) at 0, 24, 48, 72, and 96 hours after cardiac arrest in patients with good and poor 3-month neurological outcomes.

Study or Subgroup	Survivors Mean	Survivors SD	Survivors Total	Non-survivors Mean	Non-survivors SD	Non-survivors Total	Weight	Std. Mean Difference IV, Random, 95% CI
**1.1.1 Baseline**								
Einav 2013	0.003	0.001	32	0.009	0.002	126	12.7%	−3.24 [−3.77, −2.71]
Elmer 2016	0.009	0.008	39	0.125	0.091	47	12.7%	−1.70 [−2.20, −1.20]
Grubb 2007	1.793	1.673	56	3.568	2.045	77	13.0%	−0.93 [−1.29, −0.57]
Huang 2016	0.607	0.703	45	1.128	2.53	54	13.0%	−0.27 [−0.67, 0.13]
Huesgen 2021	3.7	0.91	10	3.581	1.387	12	11.7%	0.10 [−0.74, 0.94]
Petermichl 2021	2.533	0.807	21	2.291	0.638	42	12.7%	0.34 [−0.18, 0.87]
Song 2010	2.16	2.88	12	3.95	4.79	139	12.5%	−0.38 [−0.97, 0.21]
Tat 2019	0.009	0.005	12	0.061	0.028	28	11.7%	−2.15 [−2.98, −1.31]
**Subtotal (95% CI)**			**227**			**525**	**100.0%**	**−1.03 [−1.83, −0.22]**
Heterogeneity: $Tau^2 = 1.26$; $Chi^2 = 131.86$, df = 7 (P < 0.00001); $I^2 = 95\%$								
Test for overall effect: Z = 2.50 (P = 0.01)								
**1.1.2 24h**								
Elmer 2016	0.001	0.003	39	0.07	0.058	47	32.0%	−1.59 [−2.08, −1.10]
Huesgen 2021	0.083	0.009	10	1.753	1.389	12	8.0%	−1.56 [−2.54, −0.58]
Jenei 2013	0.021	0.019	24	0.109	0.105	22	19.3%	−1.17 [−1.80, −0.54]
Petermichl 2021	0.441	0.848	21	2.237	1.088	42	20.5%	−1.75 [−2.36, −1.14]
Tat 2019	0.006	0.002	12	0.081	0.047	28	12.0%	−1.85 [−2.66, −1.05]
Wiberg 2017	0.39	0.15	19	4.35	3.15	10	8.3%	−2.11 [−3.07, −1.15]
**Subtotal (95% CI)**			**125**			**161**	**100.0%**	**−1.61 [−1.89, −1.34]**
Heterogeneity: $Tau^2 = 0.00$; $Chi^2 = 3.47$, df = 5 (P = 0.63); $I^2 = 0\%$								
Test for overall effect: Z = 11.43 (P < 0.00001)								
**1.1.3 48h**								
Elmer 2016	0.002	0.001	39	0.052	0.044	47	22.5%	−1.52 [−2.01, −1.04]
Grubb 2007	0.35	0.275	56	2.568	2.298	49	24.0%	−1.39 [−1.82, −0.96]
Huesgen 2021	0.059	0.011	10	1.607	1.407	12	12.1%	−1.43 [−2.39, −0.47]
Petermichl 2021	0.272	0.473	21	2.097	1.704	42	20.1%	−1.27 [−1.84, −0.70]
Tat 2019	0.008	0.004	12	0.069	0.047	28	15.7%	−1.51 [−2.27, −0.75]
Wiberg 2017	0.18	0.08	16	2.02	0.78	7	5.7%	−4.20 [−5.80, −2.60]
**Subtotal (95% CI)**			**154**			**185**	**100.0%**	**−1.58 [−2.00, −1.16]**
Heterogeneity: $Tau^2 = 0.15$; $Chi^2 = 11.81$, df = 5 (P = 0.04); $I^2 = 58\%$								
Test for overall effect: Z = 7.32 (P < 0.00001)								
**1.1.4 72h**								
Akin 2021	0.169	0.049	168	1.094	0.432	83	17.0%	−3.67 [−4.09, −3.26]
Elmer 2016	0.001	0.001	39	0.009	0.004	47	16.2%	−2.61 [−3.19, −2.03]
Grubb 2007	0.165	0.11	56	1.215	1.034	13	15.4%	−2.31 [−3.04, −1.59]
Huesgen 2021	0.055	0.006	10	0.314	0.108	12	11.6%	−3.11 [−4.42, −1.79]
Petermichl 2021	0.123	0.261	21	0.668	0.39	42	16.1%	−1.53 [−2.12, −0.93]
Tat 2019	0.006	0.003	12	0.035	0.018	28	14.9%	−1.86 [−2.66, −1.06]
Wiberg 2017	0.16	0.08	16	2.37	1.04	5	8.9%	−4.40 [−6.18, −2.61]
**Subtotal (95% CI)**			**322**			**230**	**100.0%**	**−2.68 [−3.43, −1.92]**
Heterogeneity: $Tau^2 = 0.82$; $Chi^2 = 45.12$, df = 6 (P < 0.00001); $I^2 = 87\%$								
Test for overall effect: Z = 6.97 (P < 0.00001)								
**1.1.5 96h**								
Petermichl 2021	0.057	0.126	21	0.379	0.22	42	100.0%	−1.64 [−2.24, −1.04]
**Subtotal (95% CI)**			**21**			**42**	**100.0%**	**−1.64 [−2.24, −1.04]**
Heterogeneity: Not applicable								
Test for overall effect: Z = 5.34 (P < 0.00001)								

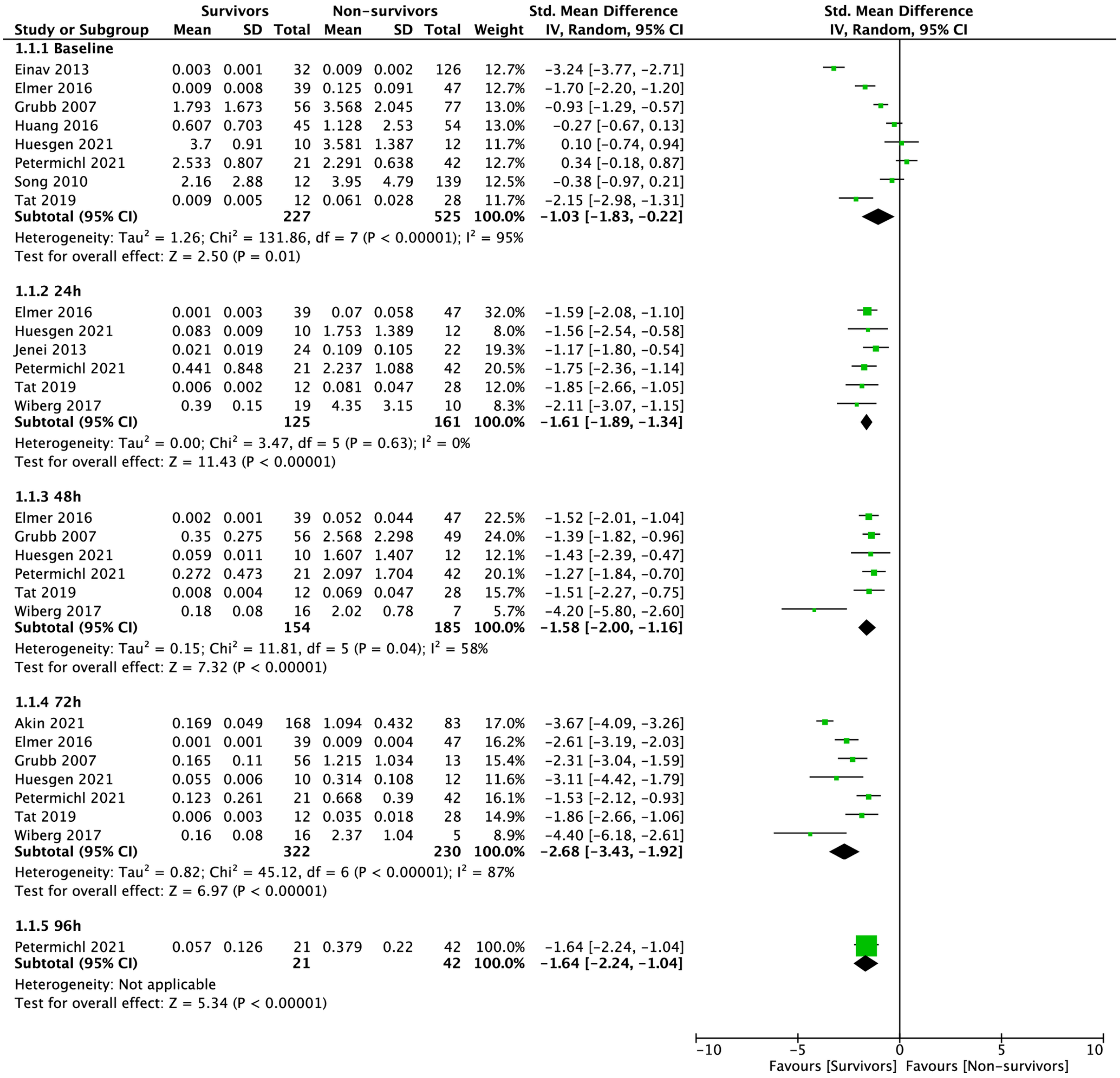

Figure S5. Forest plot presenting standardized mean differences in S100B levels between patients who survived vs. non-survived to hospital discharge at baseline and at 24, 48, 72, and 96 hours after cardiac arrest.

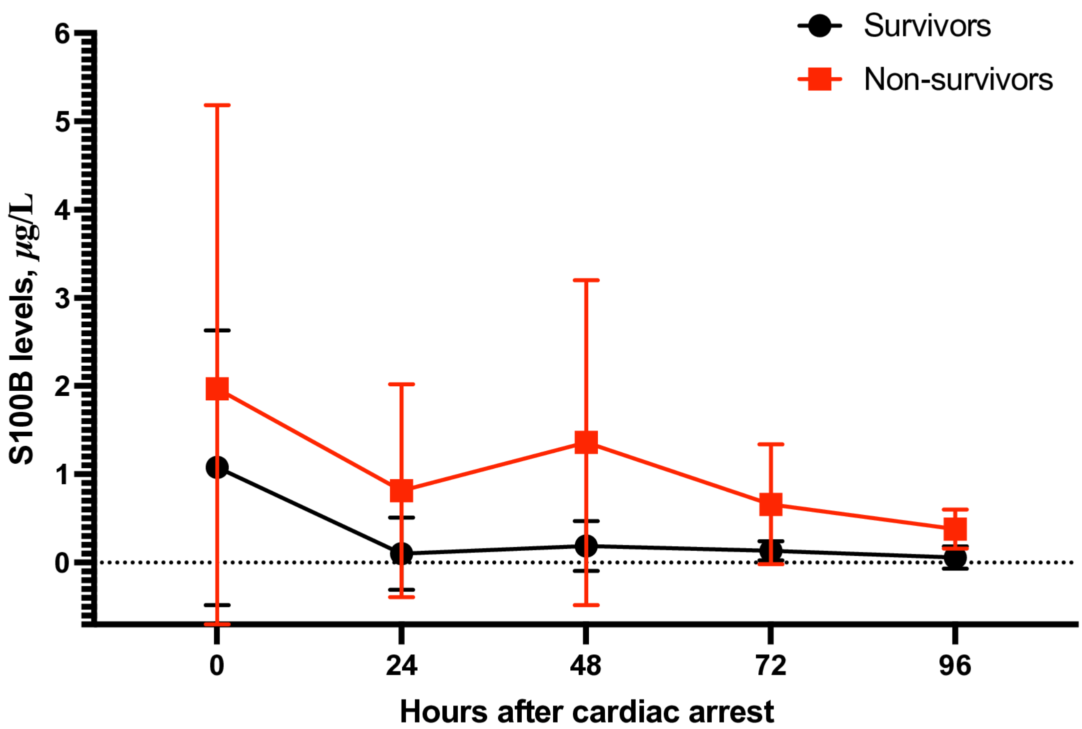

Figure S6. S100B levels (mean ± SD) at 0, 24, 48, 72, and 96 hours after cardiac arrest in patients who survived vs. non-survived to hospital discharge.

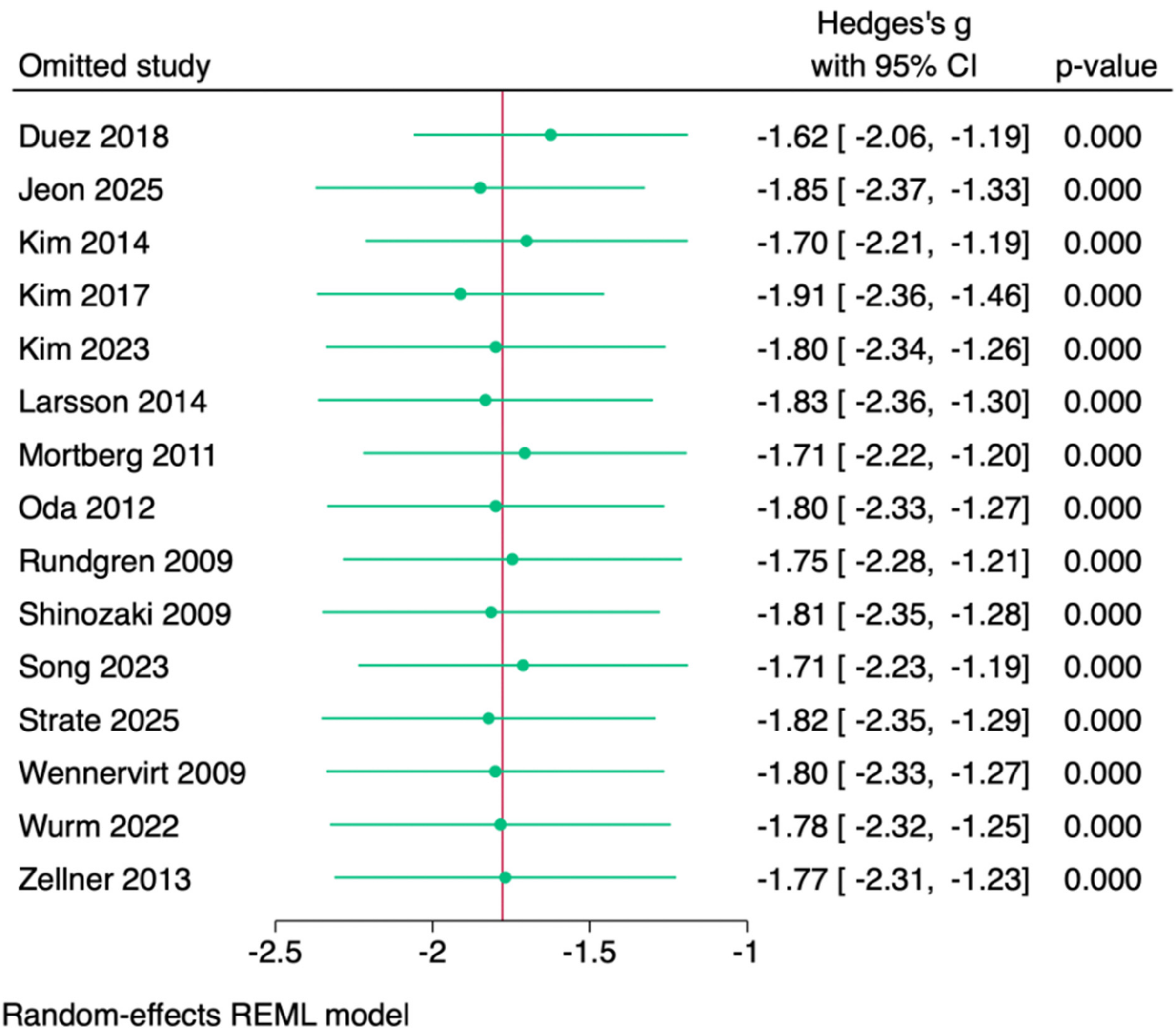

Figure S7. Leave-one-out sensitivity analysis for the primary outcome, showing the influence of each study on the pooled effect size (Hedges's g) for differences in S100B levels between outcome groups.

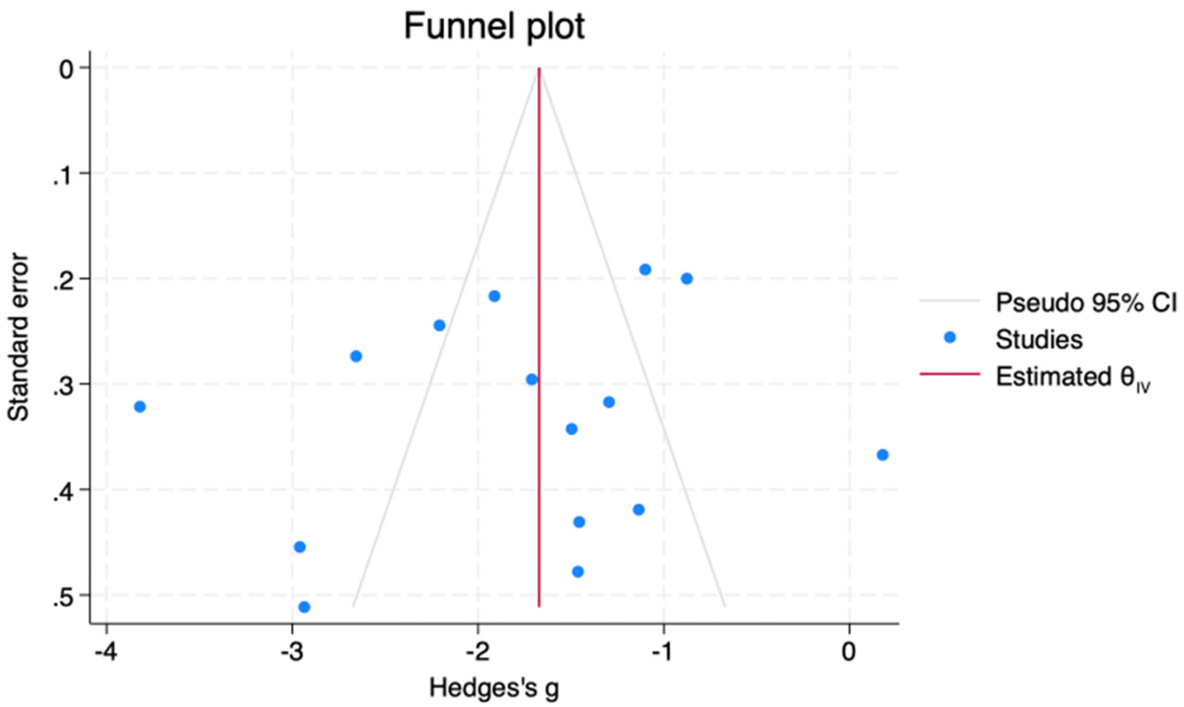

Figure S8. Funnel plot for publication bias assessment in the primary outcome.
